# Tracking hydrothermal infiltration within light oil reservoirs using organic geochemical proxies in the Shunbei area of Tarim Basin in China

**DOI:** 10.1038/s41598-025-85485-2

**Published:** 2025-01-12

**Authors:** Jian Gao, Shaojie Li, Huan Li, Zicheng Cao, Huili Li, Feng Geng, Jun Han, Cheng Huang

**Affiliations:** 1 Petroleum Exploration and Production Research Institute, SINOPEC, Beijing, 102206 China; 2 SINOPEC Key Laboratory of Geology and Resources in Deep Stratum, Beijing, 102206 China; 3 School of Geosciences, Yangtze University, Wuhan, 430100 China; 4 School of Geography, Earth and Atmospheric Sciences, The University of Melbourne, Melbourne, VIC 3010 Australia; 5 Northwest Oilfield Company, SINOPEC, Ürümqi, 830011 China

**Keywords:** Tarim Basin, Carbonate reservoir, Deep petroleum resources, Hydrothermal infiltration, Shunbei Oilfield, Geochemistry, Geology

## Abstract

Deep oil reservoirs are becoming increasingly significant fields of hydrocarbon exploration in recent decades. Hydrothermal fluid flow is deemed as a potentially crucial factor affecting the occurrence of deep oil reservoirs, such as enhancing porosity/permeability of reservoirs, accelerating oil generation and thermal cracking, and modifying organic properties of crude oils. Understanding the interplay between hydrothermal fluids and crude oils would provide useful constraints for reconstructing hydrocarbon accumulation processes and predicting the distribution patterns of crude oils. Voluminous crude oils have been discovered in the deeply buried Ordovician carbonate reservoirs within the Shunbei area of the northern Tarim Basin. Previous studies revealed that the Early Permian Tarim Large Igneous Province (LIP) has affected the Shunbei area, whereas it is still debated whether the LIP-related hydrothermal infiltration affected hydrocarbons within the Ordovician reservoirs. To resolve this puzzle, this study was designed to unravel the potential thermal impact of hydrothermal infiltration on hydrocarbons according to molecular and stable carbon isotopic compositions of oils and associated natural gases, reflectance analysis of solid bitumen, and fluid inclusion thermometry. The studied crude oils are characterized by uniform organic indicators of paraffin, terpanes, steranes, and light hydrocarbons, implying that crude oils are derived from the same source rock. Genetic binary diagrams, such as dibenzothiophene/phenanthrene (DBT/P) vs. Pr/Ph (pristane/phytane), Pr/*n*-C_17_ alkane vs. Ph/*n*-C_18_ alkane, C_31_R/C_30_hopane vs. C_26_/C_25_tricyclic terpane (TT), and C_24_/C_23_ TT vs. C_22_/C_21_ TT, indicate that marine shales deposited in a reducing-weakly oxidized environment are major source rocks. Natural gases are associated with oil reservoirs and are mainly generated via the decomposition of kerogen and crude oil. Solid bitumen with abnormally high reflectance values (2.17–2.20%) occurred in the studied area, suggesting their formation temperatures were 252–254 °C. The abnormally high temperatures may be caused by hydrothermal infiltration related to the Tarim LIP. Hydrothermal infiltration is supported by the presence of high contents of CO_2_ (30–48%) with enriched δ^13^C ratios (between − 2.5‰ and − 2.3‰), enriched *n*-alkane δ^13^C ratios, and incongruent temperatures estimated by multiple indicators, such as light hydrocarbon compositions, homogenization temperatures of fluid inclusions, and bitumen reflectance. Outcomes of this study support the interpretation that hydrothermal infiltration indeed occurred and may have facilitated hydrocarbon generation in the Shunbei area, and possibly elsewhere in the cratonic regions of the northern Tarim Basin.

## Introduction

Recent decades have witnessed a great number of oil reservoirs discovered in deep strata worldwide, and hydrothermal infiltration is increasingly recognized as an important process affecting the distribution patterns and formation process of deep oil reservoirs^1–8^. Hydrothermal fluid may affect deep oil reservoirs via multiple paths, such as enhancing porosity/permeability of reservoirs via preferential dissolution of authigenic minerals^9^, accelerating oil generation and thermal cracking^10–12^, and modifying organic properties of crude oils^13,14^. Recognizing the features of hydrothermal infiltration provides useful knowledge of the evolutionary history of deep oil reservoirs. Hydrothermal infiltration can result in a sharp increase in reservoir temperatures, which may alter specific organic geochemical features of hydrocarbons, such as producing erratic bitumen reflectance values^15^, high contents of polycyclic aromatic compounds^14^, even/odd predominance of *n*-alkanes^3^, abnormal enrichment of C_19_ and C_20_ tricyclic terpanes^3,16^, and preferential loss of ^12^C in hydrocarbons^17^. Therefore, organic geochemical features of particular hydrocarbons have the potential to provide robust evidence for understanding hydrothermal infiltration scenarios in deep oil reservoirs.

**Fig. 1:**
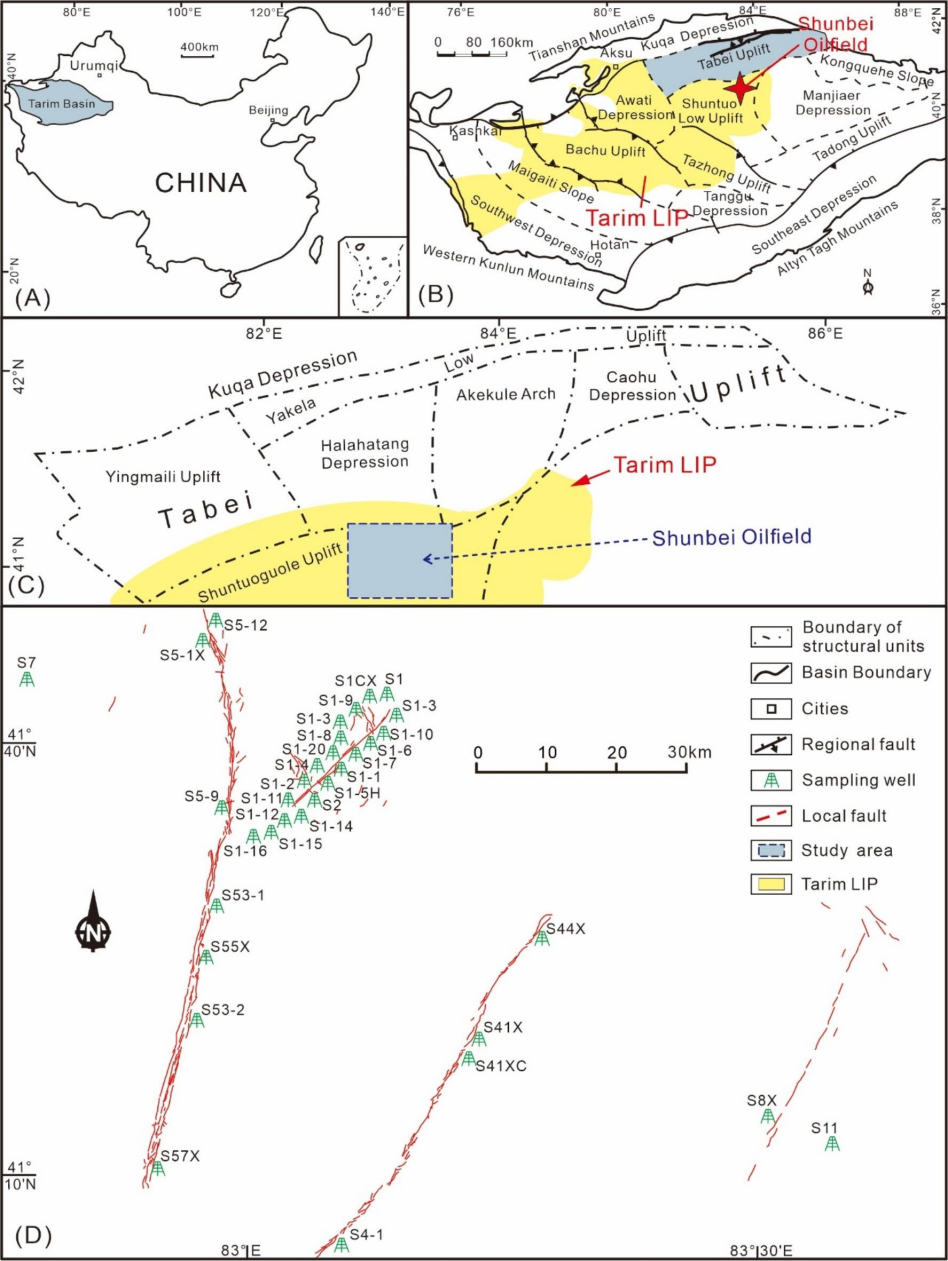
(A) Location of the Tarim Basin in northwest China; (B) simplified map showing structural units of the Tarim Basin, and distribution of Early Permian Tarim Large Igneous Province and Shunbei Oilfield; (C) location of the studied area in the northern Tarim Basin; (D) locations of sampling drill-holes in the Shunbei Oilfield. Modified after references^27,28^.

There is a suite of deeply buried oilfields discovered in the Tabei Uplift of the Tarim Basin, NW China (Fig. 1A), including the Tahe Oilfield^18^, Tuoputai Oilfield^19^, Halahatang Oilfield^20^, and Shunbei Oilfield^21^. The Shunbei Oilfield is a recently discovered petroleum exploration frontier region, located in the southern part of the Tabei Uplift and is estimated to contain ~ 17  × 10^8^ tonne of oil equivalent^22^. Reservoirs within the Shunbei Oilfield are generally buried at depths of over 7000 m, and light oils and condensates are the main types of petroleum in this region^23^. Pioneering research works have been conducted concerning the geological, geochemical, and geochronological features of the Shunbei Oilfield. For example, the distribution of oils is mainly controlled by strike-slip faults in this area^24^; oils are mainly contributed by organic-rich black shales within the Lower Cambrian strata^25^; oils were likely formed by multiple stages of accumulation processes and the main stage of oil accumulation may have taken place during the Early Devonian^21^; post-accumulation alteration processes occurred in the Shunbei Oilfield (such as biodegradation, gas washing), and gas washing may be the dominant factor responsible for the presence of condensates in this region^26^. These pieces of knowledge indeed improve the understanding of oil evolution history and exploration works in the Shunbei Oilfield.

Many past studies suggested that paleo-volcanic activities and associated hydrothermal infiltration have affected the Tarim Basin, such as the Early Permian Tarim Large Igneous Province (LIP; Fig. 1B)^3,29^. In addition to the presence of magmatic rocks (e.g. diabase) and hydrothermal minerals (e.g. calcite, pyrite, fluorite) in the strata^3,30^, molecular and isotopic compositions of reservoired hydrocarbons are reported to be altered, especially those nearby magmatic rocks and/or hydrothermal minerals^2,3,26^. Indeed, Permian dacite and volcanic tuff beds and Ordovician diabase intrusions are intersected by drillholes at 4500–5000 and 6900–6950 m, respectively in the Shunbei Oilfield^31^. This provides the direct evidence that magmatic processes have occurred in this region. However, it is still debated whether magmatic and associated hydrothermal fluid flows altered hydrocarbons in this region. For example, based on the moderate thermal maturity levels suggested by reflectance values of solid bitumen and molecular compositions of saturated and aromatic hydrocarbons (vitrinite reflectance equivalents (VR) < 1.3%), it has been proposed that the thermal impact of magmatic and hydrothermal infiltration is negligible^21^. Whereas, oils from the Shunbei Oilfield display enrichments of polycyclic aromatic hydrocarbons, which may be combustion products of saturated hydrocarbons during hydrothermal heating^26^. These findings imply that the influence of hydrothermal infiltrations on oils in the Shunbei Oilfield still needs further investigation. To resolve this puzzle, this study is designed to unravel any potential thermal impact of hydrothermal infiltration on hydrocarbons according to molecular and stable carbon isotopic compositions of oils and associated natural gases, reflectance analysis of solid bitumen, and fluid inclusion thermometry. Furthermore, these organic geochemical data are integrated into the local geological context and utilized to understand the interplay between hydrothermal infiltration and hydrocarbon accumulation processes.

## Geological background

**Fig. 2:**
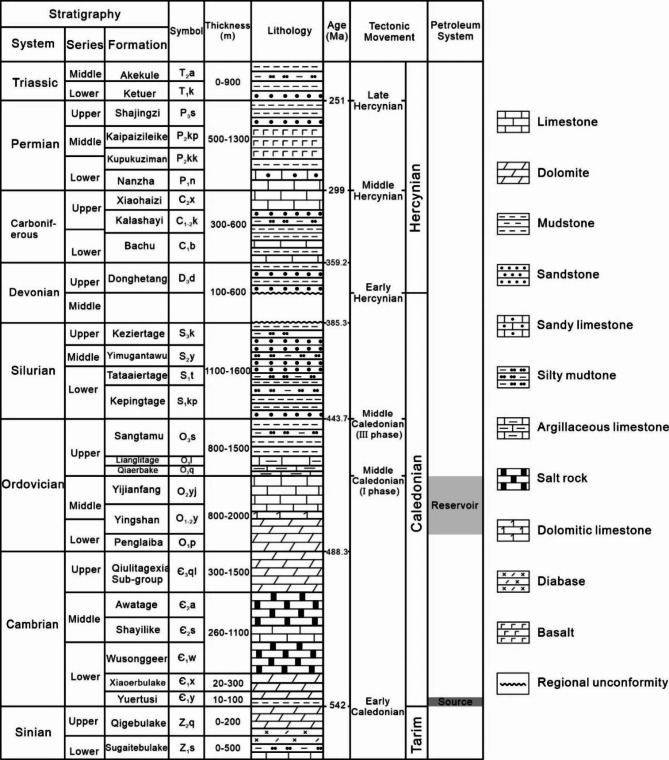
Comprehensive strata column of the Tarim Basin. Modified after reference^2^.

The Tarim Basin is one of the largest petroleum-bearing basins in northwestern China (Fig. 1A), with an area of approximately 6 × 10^5^ km^2^^32^. The basin is surrounded by mountain ranges including the Tianshan, Kunlun, and Altun mountains (Fig. 1B). Several depressions and uplifts were identified in the Tarim Basin, including the Kuqa Depression, the Awati Depression, the Munjar Depression, the Tangguizibasi Depression, the Southwestern Depression, the Southeastern Depression, the Tabei uplift, the Bachu uplift, the Tazhong uplift, and the Tanan uplift (Fig. 1B)^28,32^. The Shunbei Oilfield is located in the boundary area of Tabei Uplift and Shuntuoguole Uplift (Fig. 1C and D). The basin contains Sinian to Neogene sedimentary rocks (Fig. 2)^32^. Five evolutionary episodes were identified for the Tarim Basin, including the Sinian-Ordovician marine carbonate basin, the Silurian-Devonian marine clastic basin, the Carboniferous-Permian transitional basin, and the Mesozoic-Cenozoic continental clastic foreland basin^32^. There are multiple stages of tectonic movements that affected the Tarim Basin, such as the Caledonian, Hercynian, Indosinian, Yanshan, and Himalayan movements^33–35^. These tectonic movements also exerted crucial impacts on oil and gas accumulation processes^18,36–39^.

In the cratonic regions of the Tarim Basin, such as Tazhong Uplift and Tabei Uplift, hydrocarbons are commonly captured by marine carbonates in the Ordovician strata^40–42^. There is a long debate on the primary source rock for Ordovician-hosted hydrocarbons between Cambrian-Lower Ordovician (Є-O_1_) and Middle-Upper Ordovician (O_2-3_) organic-rich beds^3,43^. The recent discovery of widely distributed organic-rich black shales within the Yuertusi Formation (Є_1_) in the northern Tarim Basin seems to support that Cambrian black shales are likely the dominant contributor to Ordovician-hosted hydrocarbons^44^. In the Shunbei Oilfield, light oils and condensates are the main types of petroleum, and there are also other types of hydrocarbons occurring in this area including natural gases and solid bitumen^21,25,26^. The co-occurrence of multiphase hydrocarbons implies that complex petroleum accumulation processes have took place in the Shunbei Oilfield, such as secondary cracking, gas washing, biodegradation, etc^26^.

## Samples and analytical methods

### Crude oil, solid bitumen, natural gas, and reservoir rock samples

Thirty-three crude oil, three solid bitumen, and eight associated gas samples were taken from the Ordovician reservoirs of the Shunbei Oilfield (Fig. 1D). Molecular and stable carbon isotopic compositions of crude oil, organic extracts of solid bitumen, and natural gas samples were analyzed. Reflectance features of solid bitumen were also determined. Additionally, calcite veinlets occur across solid bitumen samples, and fluid inclusion thermometry analysis was conducted for calcite veinlets.

### Methodology

#### Gas chromatography-flame ionization detection (GC-FID)

The gas chromatography-flame ionization detection (GC-FID) approach was used to understand the molecular features of whole oils and light hydrocarbons. The GC-FID analysis was conducted with an Agilent 7890 A GC fitted with an HP-5 elastic quartz capillary (30 m × 0.25 mm × 0.25 μm). The GC oven was initially set at 100 °C for 1 min, and then programmed with a rate of 4 °C/min to 300 °C, holding for 25 min.

#### Organic matter extraction, liquid chromatography, and gas chromatography-mass spectrometry (GC-MS)

Molecular compositions of saturated and aromatic hydrocarbons in crude oil and solid bitumen samples were determined using gas chromatography-mass spectrometry (GC-MS). Before GC-MS analysis, soluble organic matter was extracted from solid bitumen samples, and then the soluble organic matter, together with crude oil samples, was separated into four fractions using liquid chromatography (saturates, aromatics, resins, and asphaltenes). Approximately 20 g of solid bitumen was ground into powders for microwave-assisted organic matter extraction. Powders of each sample were placed in an extraction vessel with 50 mL of dichloromethane (DCM) + methanol solvent mixture (9:1, v/v). The microwave extraction used a temperature program of 80 °C for 15 min after a ramp of 8 °C/min. After microwave extraction, the sample was centrifuged and solvent was transferred to a clean bottle containing activated copper for removing elemental sulphur present in the sample. Sulfur-removed extract was further filtered by an activated MgSO_4_ column (5.5 cm×0.5 cm). The extract was then dried and re-dissolved in a clean beaker containing DCM and activated silica gel. The silica gel/extract powder mixture was loaded onto an activated silica gel column (5.5 cm×0.5 cm) for compositional fractionation after evaporating DCM. Soluble organic matter and crude oils were further separated into four fractions using liquid chromatography. Asphaltene fractions of crude oils were collected by precipitating with *n-*heptane at room temperature for ~ 8 h (1 g sample/40 ml solvent). The remaining maltenes were further separated into saturated, aromatic, and resin fractions using alumina columns with a solvent eluting procedure (*n*-hexane, toluene, and chloroform). Glassware used in this study was firstly rinsed with methanol containing diluted HCl (0.01 N), and soaked in diluted HNO_3_ (0.01 N) overnight. Thereafter, glassware was washed with soap water and rinsed with distilled water, and dried completely before the experiment.

The GC-MS analysis of saturated fractions was performed with an HP5973 MSD connected to an HP6890 gas chromatograph. The GC was equipped with a HP-5MS capillary column (60 m × 0.25 mm × 0.25 μm). The GC oven was programmed from 50 ℃ to 310 ℃ at the rate of 3 ℃/min, with the initial and final hold times of 1 min and 30 min, respectively. The MS was operated under the conditions of source temperature of 180 ℃, ionisation energy of 70 eV and electron multiplier voltage of 1800 V. The saturated fractions were scanned in both a full scan mode (mass range: 50 –550 amu) and a selective ion monitoring (SIM) mode (mass to charge ratio, *m*/*z* = 83, 97, 123, 149, 163, 177, 183, 191, 205, 217, 218, 231, 259, 258, 372, 386, 400, 414). The aromatic fractions were scanned in a full scan mode (mass range: 50 − 550 amu). Saturated and aromatic hydrocarbons were identified through comparisons with the mass spectra and retention times of reference compounds in previous publications^45–48^. Molecular ratios were calculated using integration areas of compound peaks on the HP ChemStation Data Analysis software.

#### Carbon isotopic analysis of oil and individual N-alkanes

Stable carbon isotopic analysis was performed for whole oils and individual *n*-alkane compounds. The stable carbon isotopic ratios (δ^13^C) of whole oils were determined using a Finnigan MAT Delta Plus XLDELTA plus XL C003 mass spectrometer. The δ^13^C ratios of individual *n*-alkanes were determined using the compound-specific carbon isotope analysis (CSCIA) method. CSCIA of individual *n*-alkanes was performed on a Micromass IsoPrime isotope ratio mass spectrometer coupled with a gas chromatograph identical to what was used for GC-MS analysis. The GC was equipped with an HP-5MS capillary column (60 m × 0.25 mm × 0.25 μm). The GC oven was programmed from 50 to 310 ℃ at the rate of 3 ℃/min, with the initial and final hold times of 1 min and 10 min, respectively for the external organic reference compounds. The samples were injected via a pulsed-splitless mode (the injection was held at 15 psi above the head pressure of the column for 30 s and the purge time was 35s), and the flow rate was 1 ml/min. The GC oven was programmed from 40 to 300 °C (3 °C/min) with initial and final hold times of 1 and 30 min, respectively. Carbon isotopic ratios of samples were reported in the δ notation (δ^13^C, ‰) and relative to a CO_2_ reference gas standard (calibrated to Vienna Peedee Belemnite, VPDB). At least two replicate analyses were conducted on the same sample and the average value of the analyses is reported when the standard deviation is less than 0.4‰. A mixture of standards with known δ^13^C values was analyzed before/after each analytical session to monitor the performance of the instrument.

#### Molecular, stable carbon and hydrogen isotopic compositions of natural gases

Molecular compositions of natural gas samples were determined by a GC (CP 3800, SpectraLab Scientific) fitted with a Poraplot Q capillary column (30 m × 0.25 mm × 0.25 μm). At the initial stage, the GC oven was set at 70 °C, holding for 6 min, and then the oven was programmed to 180 °C with a rate of 15 °C/min. Specific gas species were identified with an external standard method. The stable carbon isotopic compositions of gases were determined with a Finnigan MAT-253 mass spectrometer. Similar to oil samples, carbon isotopic ratios of gases are reported in “δ” notation as per mil (‰) deviations from the δ^13^C value of VPDB. The stable hydrogen isotopic analysis was conducted using a gas chromatography-thermal conversion-isotope ratio mass spectrometry (GC-TC-IRMS, DELTA v advantage). Hydrogen isotopic ratios are reported in “δ” notation as per mil (‰) deviations from the δD value of the Vienna Standard Mean Ocean Water (VSMOW). The reproducibilities of carbon and hydrogen isotopic results were commonly less than ± 0.3‰ and ± 5‰, respectively. Standards with known δ^13^C and δD values were analyzed before and after each session to monitor the instrument’s performance.

#### Reflectance measurement of solid bitumen

The reflectance features of solid bitumen were determined following the recommendations of the ASTM D7708 (2015) standard. Random reflectance measurements were performed with a reflected light (λ = 546 nm) using a fixed microscope stage with an oil immersion objective of 50x and an ultra-fine pixel size (0.3 μm) probe. The microscope was calibrated with 2 standards, a YAG standard (0.908%) and a black standard (0.0%).

To assist in the recognition of organic matter properties, fluorescent light excitation was used.

#### Fluid inclusion thermometry

To conduct fluid inclusion microscopic analysis, double polished thin sections were made for calcite samples. Fluid inclusions were observed with a Nikon LV100 microscope which is equipped with transmitted white and incident ultraviolet light sources. Fluorescence spectrums of oil inclusions in calcites are recorded using a Nikon LV100 microscope-mounted Maya 2000Pro micro fluorescence spectrometer. Fluid inclusion microthermometry was conducted using a Zeiss Axiovert 200 M microscope equipped with a Linkam THMS600 heating-freezing stage, which had been calibrated by synthetic CO_2_ and pure H_2_O fluid inclusions. Homogenization temperature was measured with heating rates of 10 ℃/min and 5 ℃/min at low- (≤ 50 ℃) and high-temperature (> 50 ℃) conditions, respectively. The final ice melting temperature was determined using a heating/cooling rate of 1 °C/min. Uncertainties for homogenization temperatures and final ice melting temperatures are ± 1 ℃ and ± 0.1 ℃, respectively.

## Results

### Molecular compositions of paraffin, biomarker, aromatic, and light hydrocarbons

**Fig. 3:**
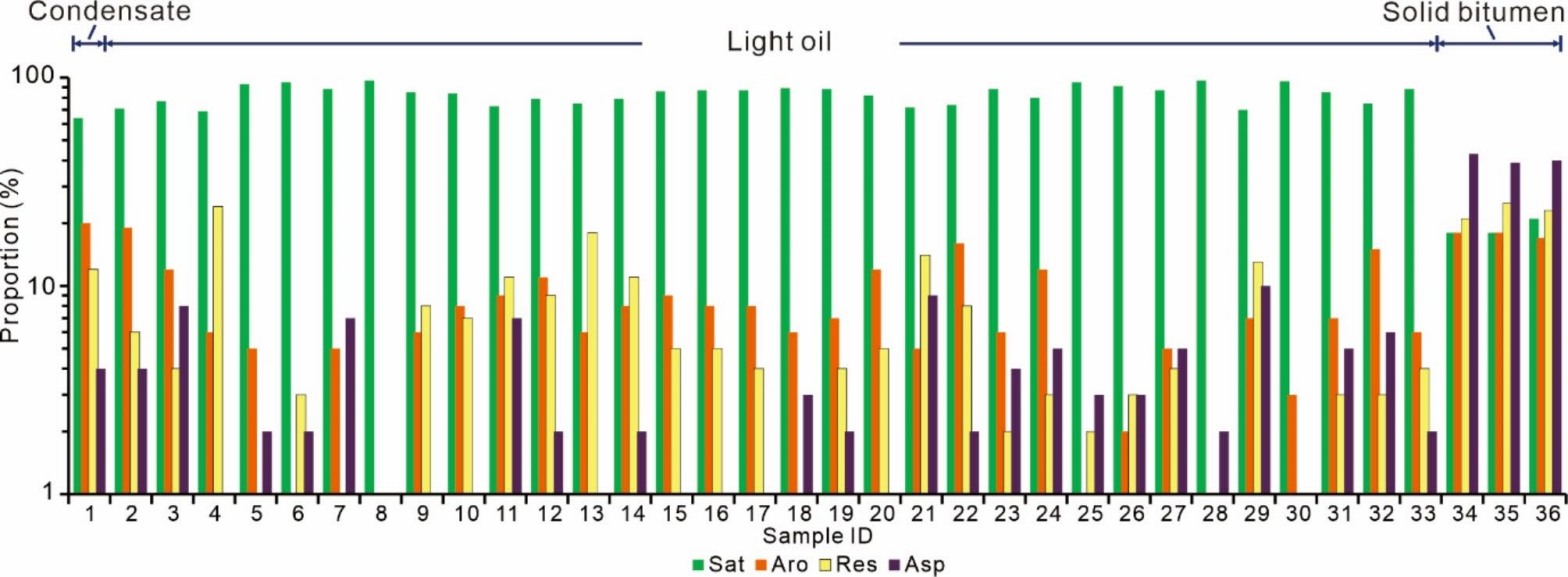
Histograms of proportions of different oil organic fractions in the studied samples. Sat, Aro, Res, and Asp denote saturated, aromatic, resin, and asphaltene fractions, respectively.

**Fig. 4:**
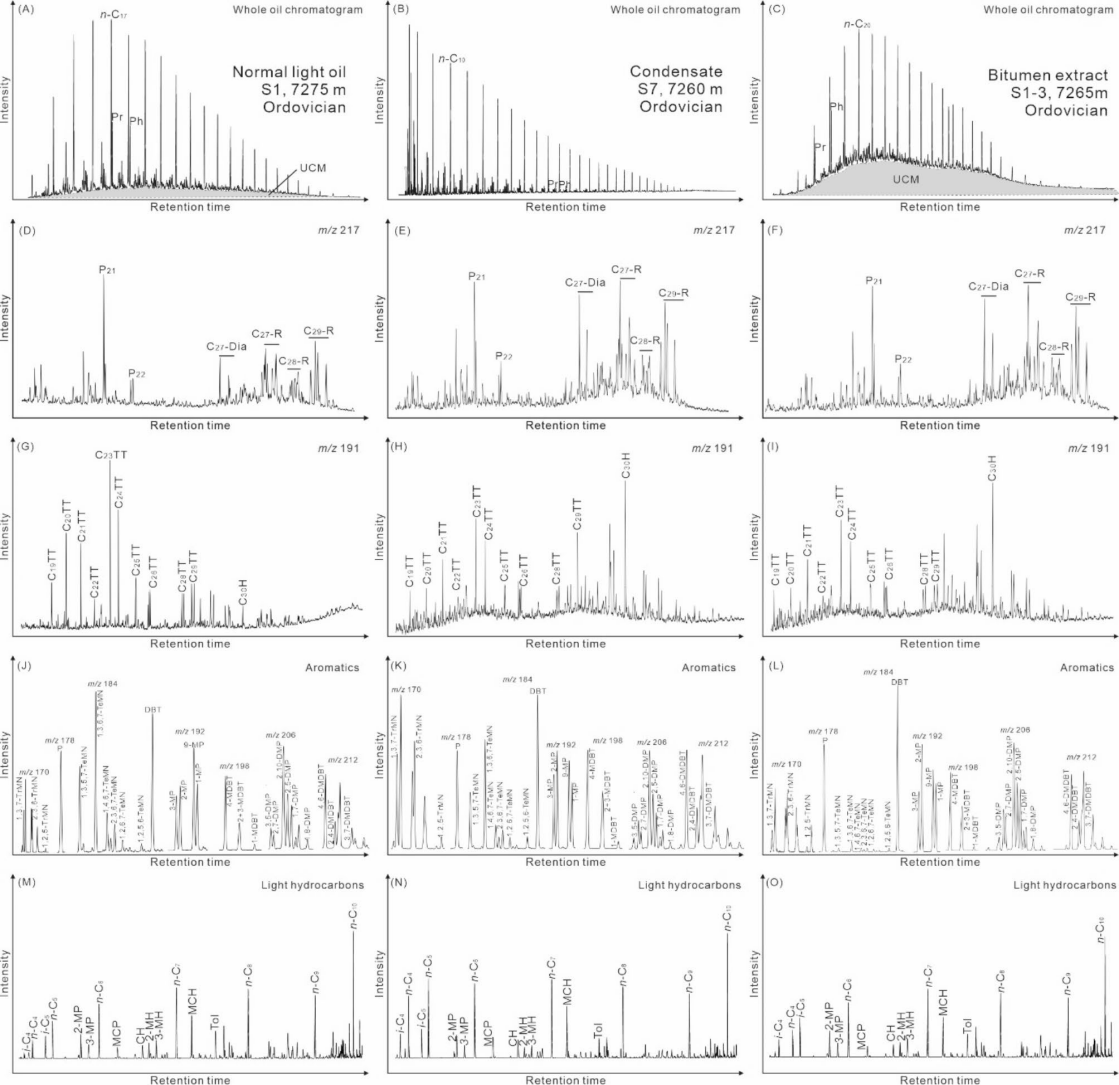
Typical chromatograms of light oils, condensate, and organic extract of solid bitumen. (A–C) whole oil chromatograms. *n*-C_x_ denotes normal alkane where x refers to carbon number. Pr and Ph denote pristane and phytane, respectively. UCM denotes “unresolved complex mixture” hump; (D-F) *m/z* 217. P_21_ and P_22_ denote C_21_ pregnane and C_22_ pregnane, respectively. C_27_-Dia denotes C_27_-diasterane. C_27_-R, C_28_-R, and C_29_-R denote C_27_ regular steranes, C_28_ regular steranes, and C_29_ regular steranes, respectively; (G-I) *m/z* 191. TT denotes tricyclic terpane. H denotes hopane; (J–L) aromatic hydrocarbons. TrMN, P, TeMN, DBT, MP, MDBT, DMP, and DMDBT denote trimethylnaphthalene, phenanthrene, tetramethylnaphthalene, dibenzothiophene, methylphenanthrene, methyldibenzothiophene, dimethylphenanthrene, dimethyldibenzothiophene, respectively. “Aromatics” refers to aromatic compound homologous series analyzed in this study; (M-O) total ion chromatogram (TIC) of different light hydrocarbons analyzed in this study. *i*-C_x_ and *n*-C_x_ denote isoalkane and normal alkane, respectively, where x refers to carbon number. MP, MCP, CH, MH, MCH, and Tol denote methylpentane, methylcyclopentane, cyclohexane, methylhexane, methylcyclohexane, and toluene, respectively.

This study investigated three main types of oils from the Shunbei Oilfield, including light oils, condensates, and oils adsorbed by solid bitumen. The saturated fraction is the dominant hydrocarbon group in light oils and condensates (64–97%; Table 1), yet oils adsorbed by solid bitumen are relatively more depleted in saturated fractions (18–21%; Fig. 3). All these three types of samples display unimodal distribution patterns in *n*-alkane series (Fig. 4A and C). The co-existence of intact *n*-alkanes and UCM (“unresolved complex mixture”) occurs in light oils and oils adsorbed by solid bitumen (Fig. 4A and C), yet there is no obvious UCM hump among the chromatogram of condensates (Fig. 4B). Samples generally have the maxima in middle-chain *n*-alkanes (*n*-C_10_ to *n*-C_23_), with (*n*-C_21_ + *n*-C_22_)/(*n*-C_28_ + *n*-C_29_) ratios are in the range of 1.8–10.5 (Table 2). The odd/even carbon number preference is negligible from *n*-alkanes of studied samples, with the Carbon Preference Index (CPI) ranges from 1.0 to 1.1 (Table 2). The ratios of pristane over phytane (Pr/Ph) fluctuate between 0.3 and 2.0 (Table 2).

Biomarkers such as steranes and terpanes are detected in the studied samples. Abundant pregnanes were identified in the samples, especially in light oils (Fig. 4D and F). In terms of regular steranes, the relative proportions of C_27_, C_28_, and C_29_ regular steranes are in the ranges of 24–49%, 19–44%, and 18–52%, respectively, and the ratios of C_28_/C_29_ regular steranes range from 0.4 to 1.9 (Table 2). There are also abundant tricyclic terpanes (TT) and hopanes (H) in the studied samples (Fig. 4G and I). The C_24_/C_23_ TT, C_22_/C_21_ TT, and C_26_/C_25_ TT ratios of samples are in the ranges of 0.4–0.7, 0.1–0.8, and 0.4–1.2, respectively (Table 2). The C_31_R/C_30_H and Gammacerane index are in the ranges of 0.2–1.9 and 0.7–5.2, respectively (Table 2). Terpane thermal maturity proxies are also in the similar ranges, including C_31_22S/(22 S + 22R)H (0.2–0.6), C_30_moretane/C_30_H (0.1–0.5), Ts/(Ts + Tm) (0.2–0.8), and C_29_Ts/(C_29_Ts + C_29_H) (0.1–0.6; Table 2). Aromatic hydrocarbon species were identified in the studied samples (Fig. 4J and L), such as trimethylnaphthalene (TMN), phenanthrene (P), tetramethylnaphthalene (TeMN), dibenzothiophene (DBT), methylphenanthrene (MP), methyldibenzothiophene (MDBT), dimethylphenanthrene (DMP), and dimethyldibenzothiophene (DMDBT). Aromatic indicators display slight variations in the studied samples (Table 3), such as DBT/P (0.01–1.72), MPI (Methylphenanthrene Index, 0.73–2.69), MPDF (methylphenanthrene distribution fraction, 0.40–0.65), MPR (methyl phenanthrene ratio, 0.92–2.20) DMP (Dimethylphenanthrene Ratio, 0.25–0.62), DMP2 (Dimethylphenanthrene Ratio 2, 0.48–0.84), MDBT (Methyldibenzothiophene Ratio, 0.68–0.98), DMDBT (Dimethyldibenzothiophene Ratio, 0.62–1.00), TrMN (Trimethylnaphthalene Ratio, 0.13–0.69), TeMN (Tetramethylnaphthalene Ratio, 0.39–0.96), and TeMN2 (Tetramethylnaphthalene Ratio 2, 0.69–1.00).

**Table 1:** Sample information and proportions of organic fractions.

Sample ID	Drillhole	Types	Depth/m	Age	Sat/%	Aro/%	Res/%	Asp/%
1	S7	Condensate	7260	Ordovician	64	20	12	4
2	S1	Light oil	7275	Ordovician	71	19	6	4
3	S1-1	Light oil	7458	Ordovician	77	12	4	8
4	S1-2	Light oil	7469	Ordovician	69	6	24	1
5	S1-3	Light oil	7256	Ordovician	93	5	1	2
6	S1-4	Light oil	7459	Ordovician	95	0	3	2
7	S1-5	Light oil	7474	Ordovician	88	5	0	7
8	S1-6	Light oil	7288	Ordovician	97	1	1	1
9	S1-7	Light oil	7339	Ordovician	85	6	8	1
10	S1-8	Light oil	7284	Ordovician	84	8	7	1
11	S1-9	Light oil	7128	Ordovician	73	9	11	7
12	S1-10	Light oil	7238	Ordovician	79	11	9	2
13	S1-11	Light oil	7157	Ordovician	75	6	18	1
14	S1-12	Light oil	7354	Ordovician	79	8	11	2
15	S1-14	Light oil	7255	Ordovician	86	9	5	0
16	S1-15	Light oil	7198	Ordovician	87	8	5	0
17	S1-16	Light oil	7210	Ordovician	87	8	4	0
18	S1-20	Light oil	7189	Ordovician	89	6	1	3
19	S1CX	Light oil	7259	Ordovician	88	7	4	2
20	S2	Light oil	7210	Ordovician	82	12	5	1
21	S4-1	Light oil	7199	Ordovician	72	5	14	9
22	S5-1X	Light oil	7208	Ordovician	74	16	8	2
23	S5-9	Light oil	7221	Ordovician	88	6	2	4
24	S5-12	Light oil	7198	Ordovician	80	12	3	5
25	S8X	Light oil	7280	Ordovician	95	0	2	3
26	S11	Light oil	7215	Ordovician	91	2	3	3
27	S41X	Light oil	7183	Ordovician	87	5	4	5
28	S41XC	Light oil	7231	Ordovician	97	0	1	2
29	S44X	Light oil	7188	Ordovician	70	7	13	10
30	S53-1	Light oil	7235	Ordovician	96	3	0	1
31	S53-2	Light oil	7190	Ordovician	85	7	3	5
32	S55X	Light oil	7180	Ordovician	75	15	3	6
33	S57X	Light oil	7150	Ordovician	88	6	4	2
34	S1-3	Solid bitumen	7265	Ordovician	18	18	21	43
35	S1-3	Solid bitumen	7266	Ordovician	18	18	25	39
36	S1-3	Solid bitumen	7270	Ordovician	21	17	23	40

Sat, Aro, Res, and asp denote saturated fraction, aromatic fraction, resin fraction, and asphaltene fraction, respectively.

**Table 2:** Molecular proxies of paraffin, terpane, and sterane compounds.

No.	A	B	C	D	E	F	G	H	I	J	K	L	M	*N*	O	*P*	Q	*R*	S	T	U
1	0.70	10	2.49	1.02	0.08	0.13	0.19	1.05	0.72	0.83	0.90	0.37	0.49	0.28	0.66	0.32	26	24	50	0.51	0.55
2	0.96	16	3.98	1.06	0.34	0.42	0.11	4.74	0.69	0.36	0.89	0.80	0.43	0.27	0.78	0.46	28	28	45	0.50	0.55
3	1.01	15	7.22	1.06	0.40	0.51	0.05	3.70	0.55	0.52	0.96	0.80	0.32	0.40	0.45	0.42	45	19	37	0.48	0.54
4	0.91	16	3.51	1.05	0.33	0.41	0.14	2.86	0.64	0.45	0.92	0.40	0.17	0.30	0.41	0.44	36	39	25	0.68	0.60
5	1.07	13	10.10	1.06	0.33	0.42	0.03	–	0.70	0.10	0.70	–	–	–	0.48	0.55	32	38	29	0.50	0.53
6	0.86	17	3.57	1.06	0.32	0.39	0.18	2.31	0.61	0.35	0.90	0.30	0.50	0.10	0.38	0.17	49	24	27	0.51	0.57
7	0.88	16	3.73	1.06	0.33	0.40	0.14	–	0.60	0.41	0.83	–	–	–	–	–	34	44	22	0.62	0.57
8	1.21	15	3.14	1.07	0.35	0.34	0.18	2.86	0.63	0.32	0.70	0.60	0.53	0.10	0.45	0.27	30	36	33	0.53	0.49
9	0.90	17	3.87	1.07	0.32	0.38	0.12	1.67	0.65	0.33	0.92	0.51	0.61	0.10	0.45	0.23	42	31	26	0.55	0.52
10	0.83	17	9.04	1.05	0.31	0.38	0.08	2.31	0.75	0.47	0.80	0.31	0.49	0.11	0.50	0.20	45	29	27	0.48	0.51
11	0.73	21	5.23	1.06	0.31	0.40	0.17	2.37	0.63	0.49	0.77	0.49	0.50	0.10	0.46	0.60	33	34	32	0.56	0.45
12	0.71	23	2.50	1.13	0.63	0.72	0.23	0.91	0.65	0.37	0.71	0.40	0.47	0.10	0.41	0.25	31	31	38	0.51	0.50
13	0.90	17	10.45	1.07	0.35	0.41	0.06	5.24	0.69	0.34	0.99	0.50	0.50	0.10	0.35	0.35	46	37	18	0.59	0.57
14	0.88	17	5.76	1.03	0.35	0.41	0.13	1.10	0.57	0.35	0.93	0.52	0.49	0.08	0.51	0.30	30	19	51	0.69	0.41
15	0.97	16	3.60	1.07	0.35	0.40	0.16	5.00	0.65	0.32	0.88	1.00	0.50	0.20	0.30	0.50	35	20	45	0.60	0.43
16	0.96	16	3.47	1.08	0.35	0.41	0.15	4.89	0.60	0.39	0.95	0.83	0.48	0.10	0.33	0.49	37	25	38	0.67	0.38
17	0.94	16	4.08	1.08	0.35	0.40	0.13	4.90	0.64	0.38	0.91	0.49	0.52	0.21	0.35	0.47	33	20	47	0.69	0.43
18	0.99	16	5.03	1.03	0.35	0.39	0.10	1.67	0.63	0.50	1.00	0.51	0.51	0.10	0.34	0.55	38	34	29	0.41	0.57
19	0.90	17	7.68	1.03	0.34	0.38	0.08	1.28	0.67	0.85	1.15	0.41	0.56	0.11	0.41	0.31	30	23	47	0.77	0.44
20	0.97	13	5.83	1.03	0.33	0.39	0.08	4.10	0.69	0.40	0.95	0.50	0.51	0.34	0.21	0.51	29	23	48	0.52	0.58
21	0.99	17	2.97	1.05	0.25	0.29	0.19	1.55	0.51	0.25	0.87	0.73	0.52	0.43	0.27	0.12	24	25	51	0.46	0.40
22	0.97	16	3.73	1.09	0.33	0.38	0.16	2.48	0.68	0.36	0.85	0.30	0.53	0.11	0.74	0.52	27	23	50	0.55	0.51
23	0.99	15	5.09	1.02	0.33	0.37	0.09	4.44	0.62	0.45	0.88	0.70	0.51	0.13	0.61	0.29	27	37	36	0.57	0.58
24	1.01	15	4.14	1.05	0.34	0.38	0.13	2.00	0.67	0.36	0.85	0.33	0.55	0.12	0.77	0.36	26	23	51	0.54	0.52
25	2.01	15	3.25	1.05	0.19	0.12	0.13	1.63	–	–	–	0.27	0.55	0.10	0.48	0.25	39	20	41	0.83	0.24
26	0.56	17	2.01	1.04	0.07	0.13	0.44	1.46	–	–	–	0.26	0.50	0.21	0.70	0.34	45	20	35	0.47	0.61
27	1.21	16	4.41	1.04	0.11	0.11	0.12	1.38	0.43	0.52	0.49	0.31	0.53	0.16	0.40	0.15	29	19	52	0.49	0.46
28	1.01	16	3.17	1.06	0.16	0.17	0.18	1.26	–	–	–	0.24	0.57	0.22	0.51	0.20	37	23	41	0.64	0.42
29	0.87	17	3.29	1.07	0.29	0.37	0.18	2.01	0.48	0.31	0.58	0.33	0.57	0.27	0.31	0.28	41	20	39	0.43	0.59
30	0.93	15	2.63	1.03	0.26	0.31	0.20	2.31	0.54	0.28	0.51	0.23	0.63	0.10	0.38	0.29	38	22	40	0.62	0.50
31	1.17	16	3.86	1.06	0.25	0.25	0.14	1.67	0.47	0.33	0.43	0.40	0.49	0.29	0.63	0.25	36	21	43	0.29	0.36
32	0.96	17	7.49	1.06	0.34	0.39	0.09	2.91	0.74	0.38	0.92	1.03	0.33	0.49	0.60	0.32	30	21	48	0.48	0.51
33	1.00	16	5.16	1.03	0.06	0.07	0.09	1.23	0.61	0.31	0.70	0.32	0.46	0.18	0.39	0.28	36	23	41	0.49	0.42
34	0.44	22	1.84	0.99	0.67	0.76	1.11	3.26	0.62	0.31	0.98	1.88	0.45	0.19	0.62	0.29	33	29	38	0.51	0.47
35	0.34	22	2.99	1.04	0.30	0.35	0.61	3.22	0.60	0.35	0.98	1.40	0.49	0.21	0.50	0.31	34	30	36	0.47	0.45
36	0.48	21	4.53	0.99	0.43	0.53	0.26	3.63	0.58	0.34	0.94	1.88	0.35	0.17	0.57	0.33	39	27	35	0.74	0.54

*A: Pr/Ph; B: C_max_, the maxima carbon number of *n*-alkanes; C: (*n*-C_21_ + *n*-C_22_)/(*n*-C_28_ + *n*-C_29_) alkanes; D: CPI (carbon preference index) = 0.5*((*n*-C_15_ + *n*-C_17_ + *n*-C_19_ + *n*-C_21_)/(*n*-C_14_ + *n*-C_16_ + *n*-C_18_ + *n*-C_20_)+(*n*-C_15_ + *n*-C_17_ + *n*-C_19_ + *n*-C_21_)/(*n*-C_16_ + *n*-C_18_ + *n*-C_20_ + *n*-C_22_)) alkanes; E: Pr/(*n*-C_17_ alkane); F: Ph/(*n*-C_18_ alkane); G: TAR (terrigenous/aquatic ratio)=(*n*-C_27_ + *n*-C_29_ + *n*-C_31_)/(*n*-C_15_ + *n*-C_17_ + *n*-C_19_) alkanes; H: Gammacerane index = 10*(gammacerane)/(gammacerane + C_30_ hopane); I: TT_24_/TT_23_, TT denotes tricyclic terpane; J: TT_22_/TT_21_; K: TT_26_/TT_25_; L: C_31_R/C_30_ hopanes; M: C_31_22S/(22 S + 22R) hopanes; N: C_30_ moretane/C_30_ hopane; O: Ts/(Ts + Tm); P: C_29_Ts/(C_29_Ts + C_29_hopane); Q: proportion of C_27_ regular steranes among the total of C_27_ regular steranes, C_28_ regular steranes, and C_29_ regular steranes; R: proportion of C_28_ regular steranes among the total of C_27_ regular steranes, C_28_ regular steranes, and C_29_ regular steranes; S: proportion of C_29_ regular steranes among the total of C_27_ regular steranes, C_28_ regular steranes, and C_29_ regular steranes; T: C_29_ ααα20S/(20 S + 20R) regular steranes; U: C_29_ αββ/(αββ + ααα) regular steranes.

The C_4_–C_10_ light hydrocarbons are also detected in the studied samples (Fig. 4M and O). Variations in light hydrocarbon ratios are minor (Table 4), such as 2,4/2,3-DMP (2,4-/2,3-dimethylpentane; 0.24–0.67), *n-*C_7_/MCH (*n*-heptane/methylcyclohexane; 1.22–2.62), Heptane value (36–45), Isoheptane value (1.00–4.42), 2-/3-MP (2-/3-methylpentane; 1.47–1.82), 2-/3-MH (2-/3-methylhexane; 0.73–1.36), *i*-/*n*-C_5_ (isopentane/*n*-pentane; 0.34–0.89), 3-MP/*n*-C_6_ (3-methylpentane/*n*-hexane; 0.17–0.31), K_1_ ratio ((2-methylhexane + 2,3-dimethylpentane)/(3-methylhexane + 2,4-dimethylpentane), 0.93–1.38), X ratio ((m-xylene + p-xylene)/(*n*-C_8_ alkane); 0.22–1.81), and toluene/*n*-C_7_ alkane (0.08–1.51).

### Stable carbon isotopic compositions of crude oils

**Fig. 5:**
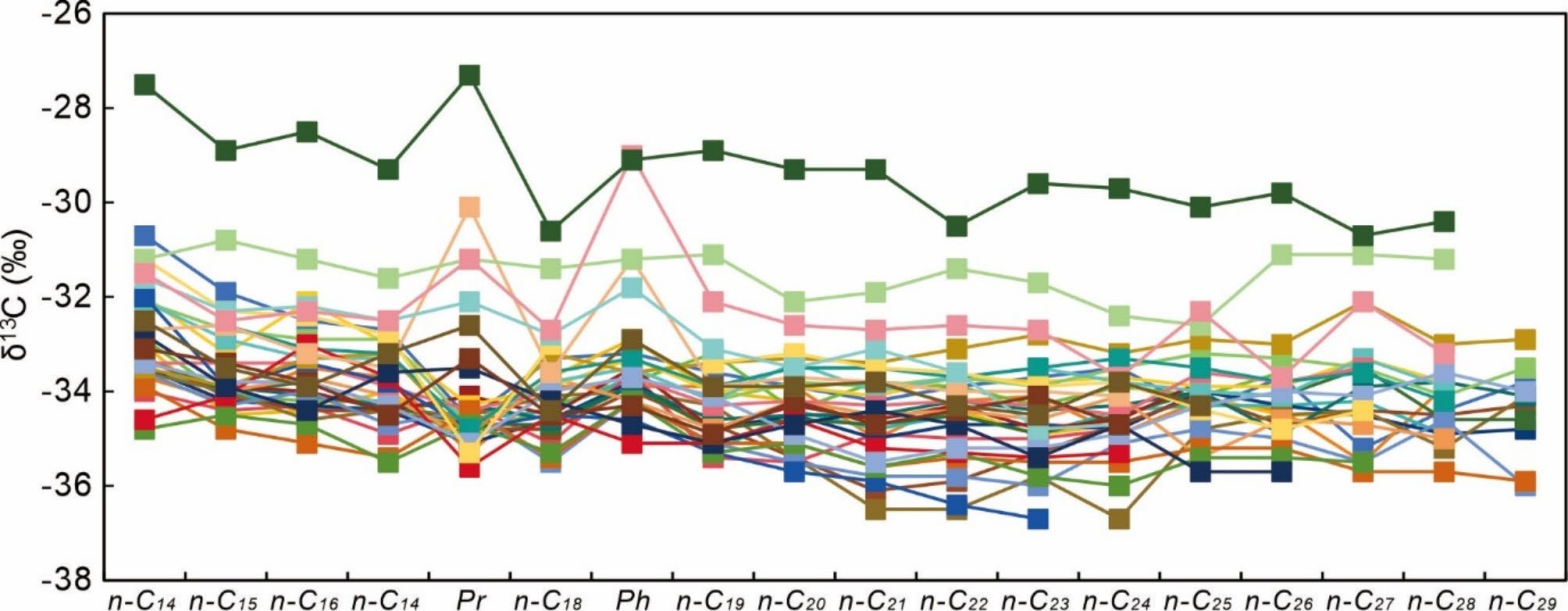
δ^13^C values of individual *n*-alkanes, Pr, and Ph compounds of studied samples.

Stable carbon isotopic compositions of bulk oils and individual *n*-alkanes are analyzed for the studied samples. The studied samples have homogeneous stable carbon isotopic compositions, with bulk δ^13^C ratios distributed between − 33.4‰ and − 29.6‰ (Table 5). The δ^13^C ratios of individual *n*-alkanes are distributed between − 36.7‰ and − 27.3‰ (Table 5). Samples broadly display uniform distribution patterns of *n*-alkane δ^13^C profiles (Fig. 5). The δ^13^C values of Pr and Ph are distributed between − 36.7‰ and − 27.3‰ and between − 35.1‰ and − 29.0‰, respectively, which are similar to those of adjacent *n*-alkanes (Fig. 5).

**Table 3:** Molecular proxies of aromatic compounds.

No	A	B	C	D	E	F	G	H	I	J	K	L	M	*N*	O	*P*
1	0.05	0.74	0.40	0.92	0.25	0.72	0.86	0.75	0.43	0.82	0.95	1.4	53.9	44.7	0.85	0.31
2	0.64	0.73	0.41	1.01	0.26	0.76	0.93	0.87	0.65	0.96	0.98	0.2	80.7	19.1	0.84	0.36
3	0.62	1.36	0.52	1.60	0.35	0.71	0.96	0.94	0.41	0.85	0.90	0.0	87.5	12.5	1.21	0.31
4	0.12	1.33	0.54	1.70	0.35	0.69	0.97	1.00	0.64	0.92	0.94	0.0	65.1	34.9	1.20	0.36
5	0.09	1.82	0.57	1.81	0.38	0.74	0.97	1.00	0.65	0.91	0.94	0.0	34.4	65.6	1.49	0.36
6	0.12	1.26	0.53	1.67	0.36	0.69	0.97	1.00	0.64	0.92	0.94	0.0	68.6	31.4	1.15	0.36
7	0.13	1.62	0.55	1.63	0.36	0.75	0.96	1.00	0.65	0.91	0.93	0.0	35.8	64.1	1.37	0.36
8	1.01	1.06	0.55	1.64	0.36	0.70	0.97	0.92	0.69	0.94	0.97	0.8	71.9	27.3	1.04	0.36
9	0.91	1.07	0.54	1.61	0.33	0.70	0.96	0.93	0.66	0.94	0.96	0.5	71.4	28.1	1.04	0.36
10	0.21	1.14	0.44	1.15	0.30	0.76	0.92	0.92	0.51	0.52	0.92	0.0	96.1	3.9	1.09	0.33
11	0.01	1.18	0.44	1.16	0.31	0.75	0.92	0.93	0.46	0.55	0.89	0.0	95.6	4.3	1.11	0.32
12	0.68	1.34	0.53	1.67	0.36	0.70	0.96	0.95	0.43	0.87	0.91	0.0	88.1	11.9	1.20	0.31
13	0.41	1.35	0.53	1.63	0.35	0.70	0.96	0.96	0.51	0.79	0.91	0.0	86.8	13.2	1.21	0.33
14	0.42	1.44	0.53	1.69	0.36	0.70	0.96	0.95	0.52	0.95	1.00	0.0	90.2	9.7	1.26	0.33
15	0.40	1.42	0.52	1.61	0.35	0.71	0.96	0.97	0.51	0.90	0.92	0.0	85.7	14.3	1.25	0.33
16	0.46	1.40	0.54	1.70	0.36	0.70	0.96	0.97	0.55	0.91	1.00	0.0	84.1	15.9	1.24	0.34
17	0.44	1.42	0.55	1.75	0.37	0.69	0.97	0.96	0.55	0.92	0.94	0.0	86.6	13.3	1.25	0.34
18	0.94	1.20	0.53	1.64	0.35	0.70	0.96	0.94	0.57	0.94	0.96	0.1	80.6	19.3	1.12	0.34
19	0.33	1.27	0.46	1.36	0.36	0.78	0.85	0.97	0.43	0.42	0.93	0.0	99.0	1.0	1.16	0.31
20	0.46	0.86	0.46	1.21	0.28	0.72	0.95	0.92	0.67	0.95	0.97	2.3	63.1	34.6	0.91	0.36
21	0.25	1.71	0.54	1.61	0.43	0.65	0.74	0.80	0.13	0.40	0.85	0.0	96.2	3.8	1.42	0.26
22	0.24	0.86	0.42	1.03	0.26	0.77	0.90	0.83	0.26	0.88	0.95	0.0	81.9	18.1	0.92	0.28
23	0.43	1.26	0.55	1.71	0.38	0.65	0.98	0.96	0.64	0.92	1.00	0.2	72.7	27.1	1.16	0.35
24	0.30	0.77	0.42	1.03	0.26	0.77	0.90	0.83	0.54	0.88	0.96	0.7	73.6	25.7	0.86	0.34
25	0.53	2.69	0.65	2.12	0.62	0.48	0.90	0.94	0.47	0.75	0.96	0.3	87.1	12.6	2.01	0.32
26	0.87	1.23	0.46	1.25	0.33	0.84	0.96	0.94	0.31	0.82	0.87	0.5	39.4	60.1	1.14	0.29
27	1.20	1.52	0.61	1.66	0.51	0.48	0.68	0.62	0.37	0.46	0.77	0.0	87.9	12.1	1.31	0.30
28	0.79	1.63	0.52	1.27	0.43	0.66	0.73	0.89	0.41	0.64	0.90	0.1	90.9	9.0	1.38	0.31
29	0.22	2.02	0.59	1.86	0.46	0.62	0.88	0.90	0.16	0.39	0.69	0.0	93.4	6.6	1.61	0.26
30	0.51	1.75	0.63	2.20	0.48	0.57	0.90	0.91	0.35	0.66	0.85	0.0	73.6	26.4	1.45	0.30
31	1.72	1.07	0.63	1.70	0.51	0.48	0.72	0.65	0.57	0.66	0.88	0.1	76.9	23.0	1.04	0.34
32	0.36	1.16	0.52	1.48	0.35	0.72	0.95	0.83	0.57	0.85	0.93	0.0	77.5	22.5	1.10	0.34
33	1.27	1.91	0.64	1.85	0.49	0.56	0.90	0.94	0.56	0.78	0.92	0.0	90.1	9.9	1.55	0.34
34	0.65	1.07	0.48	1.41	0.33	0.73	0.95	0.92	0.23	0.60	0.76	0.0	90.3	9.7	1.04	0.27
35	0.94	1.12	0.52	1.56	0.35	0.75	0.96	0.93	0.50	0.80	0.88	0.0	87.6	12.4	1.07	0.33
36	0.72	1.14	0.50	1.47	0.34	0.73	0.95	0.92	0.42	0.61	0.77	0.0	88.9	11.0	1.08	0.31

*A: DBT/P, DBT and P denote dibenzothiophene and phenanthrene, respectively; B: MPI (Methylphenanthrene Index) = 1.5*(2-MP + 3-MP)/(*P* + 1-MP + 9-MP), MP denotes methylphenanthrene; C: MPDF=(2-MP + 3-MP)/(2-MP + 3-MP + 1-MP + 9-MP); D: MPR=(2-MP/1-MP); E: DMP (dimethylphenanthrene maturity ratio)=(2,6-DMP + 2,7-DMP)/(1,6-DMP + 2,9-DMP + 1,7-DMP + 2,6-DMP + 2,7-DMP), DMP denotes dimethylphenanthrene; F: DMP2 (C_2_-alkylphenanthrene organic matter type indicator) = 1,7DMP/2-EP + 9-EP + 3,6-DMP + 1,7-DMP, EP denotes ethylphenanthrene; G: MDBT (methyldibenzothiophene ratio) = 4-MDBT/1-MDBT + 4-MDBT, MDBT denotes methyldibenzothiophene; H: DMDBT (dimethyldibenzothiophene ratio)=(4,6-DMDBT + 2,4-DMDBT)/(1,4-DMDBT + 1,6-DMDBT + 1,8-DMDBT + 4,6-DMDBT + 2,4-DMDBT), DMDBT denotes dimethyldibenzothiophene; I: TrMN (trimethylnaphthalene ratio)=(1,3,7-TrMN + 1,3,6-TrMN + 2,3,6-TrMN)/(1,3,7-TrMN + 1,3,6-TrMN + 1,4,6-TrMN + 1,3,5-TrMN + 2,3,6-TrMN + 1,6,7-TrMN + 1,2,6-TrMN + 1,2,5-TrMN), TrMN denotes trimethylnaphthalene; J: TeMN (tetramethylnaphthalene ratio)=(1,3,6,7-TeMN + 2,3,6,7-TeMN)/(1,2,3,7-TeMN + 1,2,3,6-TeMN + 1,2,5,6-TeMN + 1,3,6,7-TeMN + 2,3,6,7-TeMN), TeMN denotes tetramethylnaphthalene; K: TeMN2 (2nd tetramethylnaphthalene ratio)= (1,3,6,7-TeMN + 1,2,4,7-TeMN + 1,2,5,7-TeMN + 2,3,6,7-TeMN + 1,2,6,7-TeMN + 1,2,3,7-TeMN + 1,2,3,6-TeMN)/(1,3,6,7-TeMN + 1,2,4,7-TeMN + 1,2,5,7-TeMN + 2,3,6,7-TeMN + 1,2,6,7-TeMN + 1,2,3,7-TeMN + 1,2,3,6-TeMN + 1,2,5,6-TeMN); L: dibenzofurans (%), proportion of integration area of dibenzofurans among the total integration area of dibenzofurans, dibenzothiophenes, and fluorenes; M: dibenzothiophenes (%), proportion of integration area of dibenzothiophenes among the total integration area of dibenzofurans, dibenzothiophenes, and fluorenes; N: fluorenes (%), proportion of integration area of fluorenes among the total integration area of dibenzofurans, dibenzothiophenes, and fluorenes; O: R_MPI_ (VR calculated based on MPI) = 0.6*MPI + 0.4; P R_TrMN_ (VR calculated based on TrMN) = 0.1955* TrMN + 0.2298.

**Table 4:** Molecular proxies of light hydrocarbons.

No	A	B	C	D	E	F	G	H	I	J	K	L	M	*N*	O	*P*	Q
1	0.27	1.57	37	1.00	1.77	0.84	0.34	0.17	1.06	0.22	0.08	651,361	2.9	28	26	47	120
2	0.32	1.80	39	2.38	1.76	0.86	0.59	0.25	1.03	0.55	0.34	1,230,623	1.3	35	14	51	123
3	0.29	1.74	39	2.30	1.69	0.84	0.58	0.24	1.03	0.59	0.39	271,633	1.4	33	13	54	121
4	0.31	1.72	38	2.31	1.71	0.83	0.55	0.25	1.02	0.52	0.31	1,215,544	1.3	35	14	51	123
5	0.34	1.77	38	2.44	1.78	0.88	0.62	0.26	1.05	0.55	0.36	970,041	1.2	36	14	51	124
6	0.32	1.67	37	2.25	1.74	0.84	0.59	0.26	1.02	0.55	0.33	762,130	1.3	35	14	51	123
7	0.28	1.63	38	2.22	1.64	0.81	0.55	0.24	1.01	0.56	0.35	394,756	1.4	33	14	54	121
8	0.33	1.72	37	2.33	1.79	0.86	0.65	0.27	1.04	0.59	0.38	409,584	1.3	35	14	51	123
9	0.32	1.72	37	2.29	1.76	0.85	0.62	0.27	1.04	0.59	0.37	412,449	1.3	35	14	51	123
10	0.37	1.67	36	2.32	1.80	0.88	0.64	0.27	1.05	0.51	0.35	189,598	1.3	36	14	50	125
11	0.34	1.61	36	2.25	1.77	0.86	0.65	0.27	1.04	0.59	0.40	156,035	1.3	34	14	52	124
12	0.33	1.79	38	2.39	1.82	0.87	0.64	0.26	1.04	0.56	0.36	950,764	1.3	36	14	50	124
13	0.34	1.81	38	2.35	1.80	0.84	0.62	0.26	1.02	0.51	0.29	316,166	1.3	38	15	48	124
14	0.34	1.78	38	2.32	1.77	0.83	0.58	0.26	1.01	0.52	0.30	235,322	1.3	37	15	49	124
15	0.35	1.82	38	2.36	1.81	0.84	0.64	0.27	1.01	0.51	0.29	219,920	1.2	38	15	48	124
16	0.33	1.87	39	2.42	1.77	0.83	0.57	0.25	1.01	0.48	0.27	364,772	1.2	38	14	48	124
17	0.35	1.89	39	2.48	1.79	0.84	0.58	0.26	1.01	0.48	0.27	344,616	1.2	39	14	47	124
18	0.33	1.87	39	2.43	1.78	0.86	0.62	0.26	1.04	0.52	0.32	297,427	1.2	37	14	49	123
19	0.32	1.78	38	2.38	1.79	0.86	0.61	0.26	1.04	0.53	0.34	634,551	1.3	36	14	50	123
20	0.24	1.49	37	1.86	1.67	0.77	0.53	0.24	0.99	0.55	0.34	259,702	1.6	30	15	55	119
21	0.35	2.37	43	2.71	1.60	0.97	0.64	0.26	1.13	0.69	0.55	599,858	1.1	34	12	54	124
22	0.34	1.71	38	1.94	1.79	0.83	0.52	0.23	1.02	0.35	0.21	263,286	1.4	36	17	48	124
23	0.28	1.23	41	2.28	1.47	0.73	0.88	0.24	0.93	0.87	0.78	5771	1.4	19	8	74	121
24	0.33	1.76	37	1.88	1.77	0.83	0.54	0.24	1.02	0.34	0.20	183,407	1.4	37	17	46	123
25	0.29	1.80	38	3.68	1.74	1.03	0.80	0.31	1.13	0.64	0.50	1,941,859	1.5	36	10	53	121
26	0.33	1.76	38	2.39	1.78	0.86	0.65	0.27	1.04	0.52	0.32	1,340,829	1.3	37	14	49	123
27	0.40	2.41	44	3.15	1.57	0.96	0.79	0.28	1.09	0.91	0.74	347,619	1.0	31	10	59	126
28	0.55	2.62	41	3.33	1.70	1.13	0.89	0.29	1.19	0.94	0.75	1,456,303	1.0	36	11	54	131
29	0.36	1.97	40	2.85	1.71	0.93	0.63	0.26	1.08	0.62	0.42	1,055,887	1.2	36	12	51	125
30	0.52	1.75	38	3.45	1.70	1.00	0.68	0.27	1.10	0.61	0.43	1,085,781	0.8	39	10	51	130
31	0.43	2.16	45	4.42	1.55	1.10	0.69	0.23	1.21	1.28	1.51	50,693	0.7	21	5	74	127
32	0.39	1.70	38	2.44	1.80	0.88	0.58	0.25	1.04	0.45	0.28	859,190	1.2	37	14	49	126
33	0.67	1.22	36	3.48	1.64	1.36	0.66	0.21	1.38	1.81	1.39	1,261,853	0.8	22	6	72	134
34	0.33	1.76	38	2.45	1.77	0.87	0.63	0.26	1.05	0.54	0.35	1,147,383	1.3	36	14	51	123
35	0.34	1.77	38	2.50	1.80	0.88	0.63	0.25	1.03	0.53	0.35	1,150,383	1.3	36	13	51	123
36	0.32	1.75	38	2.40	1.78	0.86	0.61	0.24	1.04	0.55	0.34	1,147,333	1.3	36	14	50	122

*A: 2,4-DMP/2,3-DMP, DMP denotes dimethylpentane; B: (*n*-C_7_ alkane)/MCH, MCH denotes methylcyclohexane; C: H value=(*n*-C_7_ alkane×100)/(CH + 2-MH + 2,3-DMP + 1,1-DMCP + 3-MH + 1,cis,3-DMCP + 1,trans,3-DMCP + 1,trans,2-DMCP + *n*-C_7_ + MCH), CH, MH, DMCP denote cyclohexane, methylhexane, and dimethylcyclopentane, respectively; D: I value=(2-MH + 3-MH)/(1,cis,3 + 1,trans,3 + 1,trans,2)-DMCP; E: 2-MP/3-MP, MP denotes methylpentane; F: 2-MH/3-MH; G: (*i*-C_5_/*n*-C_5_) alkanes; H: (3-MP)/(*n*-C_6_ alkane); I: K_1_=(2-MH + 2,3-DMP)/(3-MH + 2,4-DMP); J: X ratio=(m-xylene + p-xylene)/(*n*-C_8_ alkane); K: toluene/(*n*-C_7_ alkane); L: integration area of P_2_ + N_2_, P_2_ = 2-MH + 3-MH, N_2_= (1,1 + 1c3 + 1t3)–DMCP; M: N_2_/P_3_, P_3_ = 3-EH + 3,3-DMP + 2,2-DMP + 2,3-DMP + 3,3-DMP + 2,2,3-TMB, EH and TMB denote ethylpentane and trimethylbutane, respectively; N: proportion of integration area of 3RP among the total integration area of 3RP, 5RP, and 6RP, 3RP=(2,2-DMP + 2,2,3-TMB + 3,3-DMP + 2-MH + 2,3-DMP + 3-MH + 2,4–DMP); O: proportion of integration area of 5RP among the total integration area of 3RP, 5RP, and 6RP, 5RP=(1,1 + 1c3-DMCyC_5_ + 1t3 + 1t2)-DMCP; P: proportion of integration area of 6RP among the total integration area of 3RP, 5RP, and 6RP, 6RP = MCH + toluene; Q: temperature (℃) = 140 + 15*[ln(2,4-DMP/2,3-DMP)].

**Table 5:** Stable carbon isotopic compositions of bulk crude oils and *n*-alkanes (δ^13^C, ‰).

No	Oil	*n*-C_14_	*n*-C_15_	*n*-C_16_	*n*-C_14_	Pr	*n*-C_18_	Ph	*n*-C_19_	*n*-C_20_	*n*-C_21_	*n*-C_22_	*n*-C_23_	*n*-C_24_	*n*-C_25_	*n*-C_26_	*n*-C_27_	*n*-C_28_	*n*-C_29_
1	-29.6	– 30.7	– 31.9	– 32.5	– 32.7	– 34.6	– 33.3	– 33.2	– 33.6	– 33.9	– 34.2	– 33.9	– 33.7	– 33.5	– 34.4	– 33.7	– 35.2	– 34.4	– 33.8
2	– 32.5	– 33.7	– 34.2	– 33.5	– 33.7	– 35.3	– 33.9	-33.9	– 33.9	– 33.9	– 35.0	– 34.2	– 34.1	– 34.9	– 34.2	– 34.4	– 35.5	– 33.9	–
3	– 32.3	– 33.0	– 34.5	– 34.4	– 34.0	– 35.0	– 33.7	– 32.9	– 34.0	– 34.2	– 34.5	– 34.4	– 34.9	– 34.7	– 34.1	– 34.5	– 34.5	–	–
4	– 31.9	– 32.9	– 34.1	– 34.2	– 34.2	– 35.0	– 34.3	– 33.8	– 33.2	– 34.4	– 33.8	– 33.9	– 34.3	– 33.9	– 34.1	– 33.6	–	–	–
5	– 31.9	– 33.5	– 34.0	– 34.4	– 34.3	– 34.6	– 34.4	– 33.7	– 35.3	– 34.4	– 34.8	– 34.5	– 34.9	– 34.4	– 34.3	– 34.3	– 34.2	– 34.9	–
6	– 31.8	– 33.5	– 34.2	– 34.3	– 34.4	– 35.1	– 34.6	– 33.8	– 34.8	– 34.5	– 35.0	– 34.7	– 34.7	– 34.8	– 34.0	– 34.3	– 34.5	– 34.9	– 34.8
7	– 32	– 33.3	– 34.1	– 34.4	– 34.4	– 34.8	– 34.8	– 34.0	– 34.7	– 35.4	– 36.1	– 35.9	– 35.3	– 34.7	– 34.1	– 34.7	– 34.4	– 34.5	– 34.3
8	– 32	– 33.6	– 34.0	– 34.6	– 34.4	– 34.5	– 34.1	– 34.0	– 34.3	– 35.4	– 36.5	– 36.5	– 35.8	– 36.7	– 34.8	– 34.5	– 34.4	– 35.2	– 34.1
9	– 31.8	– 33.3	– 34.0	– 33.8	– 34.3	– 34.7	– 34.7	– 33.9	– 34.8	– 34.6	– 34.7	– 34.5	– 34.9	– 34.5	– 34.0	– 34.1	– 33.5	– 34.6	– 34.6
10	– 32	–	– 33.9	– 34.1	– 34.3	– 34.3	– 34.6	– 33.9	– 34.6	– 34.5	– 34.5	– 34.2	– 34.4	– 34.3	– 34.0	– 34.9	– 33.9	– 33.8	– 34.1
11	– 32	–	– 33.9	– 34.0	– 34.4	– 34.1	– 34.5	– 33.5	– 34.8	– 34.3	– 34.6	– 34.3	– 34.8	– 34.6	– 33.8	–	–	–	–
12	– 32.5	– 33.6	– 34.3	– 34.0	– 34.7	– 34.4	– 35.5	– 34.2	– 35.1	– 35.5	– 35.8	– 35.8	– 36.0	– 35.1	– 34.8	– 35.0	– 35.5	– 34.6	– 36.0
13	– 32.1		– 34.0	– 33.7	– 34.1	– 35.0	– 33.7	– 34.2	– 34.8	– 34.2	– 34.5	– 34.4	– 34.6	– 34.1	– 35.4	– 34.6	– 34.7	– 35.0	–
14	– 32.4	– 32.0	– 33.2	– 32.1	– 33.0	– 34.3	– 33.9	– 33.3	– 34.0	– 33.9	– 34.1	– 33.7	– 33.8	– 33.7	– 33.9	– 33.9	– 33.3	– 33.8	–
15	– 30.5	– 32.1	– 32.7	– 32.9	– 32.9	– 34.7	– 33.8	– 33.5	– 34.2	– 33.8	– 33.9	– 33.7	– 33.9	– 33.5	– 33.2	– 33.3	– 33.5	– 34.1	– 33.5
16	– 32.5	– 32.1	– 32.9	– 33.3	– 33.3	– 34.7	– 33.8	– 33.5	– 34.2	– 33.4	– 34.0	– 33.7	– 33.5	– 33.8	– 34.0	– 34.1	– 33.3	– 33.9	–
17	– 32.3	– 33.4	– 33.4	– 33.4	– 33.9	– 34.4	– 34.0	– 33.7	– 34.3	– 34.2	– 34.3	– 34.2	– 34.3	– 34.5	– 33.6	– 33.8	– 33.5	–	–
18	– 32.2	– 32.0	– 33.9	– 33.4	– 33.8	– 34.8	– 34.5	– 34.6	– 35.3	– 35.7	– 35.9	– 36.4	– 36.7	–	–	–	–	–	–
19	– 32.3	– 33.9	– 34.8	– 35.1	– 35.4	– 34.4	– 35.4	– 34.2	– 35.1	– 35.1	– 35.6	– 35.4	– 35.5	– 35.5	– 35.2	– 35.2	– 35.7	– 35.7	– 35.9
20	– 32.7	– 33.5	– 33.9	– 33.3	– 33.2	– 35.0	– 33.2	– 33.6	– 33.4	– 33.3	– 33.4	– 33.1	– 32.8	– 33.2	– 32.9	– 33.0	– 32.1	– 33.0	– 32.9
21	– 31.6	– 34.8	– 34.5	– 34.7	– 35.5	– 34.7	– 35.3	– 34.2	– 35.3	– 35.1	– 35.6	– 35.3	– 35.8	– 36.0	– 35.4	– 35.4	– 35.5		–
22	– 32		– 32.6	– 33.1	– 33.2	– 34.8	– 33.6	– 33.3	– 33.9	– 33.5	– 33.5	– 33.7	– 33.5	– 33.3	– 33.5	– 33.8	– 33.6	– 34.2	–
23	– 32.1	– 34.6	– 34.1	– 33.0	– 33.7	– 35.6	– 34.5	– 35.1	– 35.1	– 34.6	– 35.2	– 35.3	– 35.4	– 35.3	–	–	–	–	–
24	– 31.9	– 33.4	– 33.8	– 33.8	– 34.3	– 35.1	– 34.0	– 33.7	– 34.1	– 34.9	– 35.5	– 35.2	– 35.2	– 34.9	– 34.3	– 34.0	– 34.1	– 33.6	– 34.0
25	– 30.4	– 32.7	– 32.6	– 33.2	– 33.5	– 30.1	– 33.6	– 31.2	– 33.9	– 33.7	– 33.8	– 34.0	– 34.0	– 34.1	–	–	–	–	–
26	– 31.6	– 31.2	– 32.3	– 32.4	– 32.8	– 35.3	– 33.0		– 33.4	– 33.2	– 33.5	– 33.6	– 33.9	– 33.8	– 34.4	– 34.8	– 34.4		–
27	– 31.8	– 31.2	– 30.8	– 31.2	– 31.6	– 31.2	– 31.4	– 31.2	– 31.1	– 32.1	– 31.9	– 31.4	– 31.7	– 32.4	– 32.6	– 31.1	– 31.1	– 31.2	–
28	– 30.2	– 31.6	– 32.3	– 32.2	– 32.5	– 32.1	– 32.8	– 31.8	– 33.1	– 33.5	– 33.1	– 33.6	– 34.8	– 34.7	–	–	–	–	–
29	– 31.7	– 31.5	– 32.5	– 32.3	– 32.5	– 31.2	– 32.7	– 29.0	– 32.1	– 32.6	– 32.7	– 32.6	– 32.7	– 33.7	– 32.3	– 33.7	– 32.1	– 33.2	–
30	– 31.8	– 32.8	– 33.9	– 34.4	– 33.6	– 33.5	– 34.2	– 34.7	– 35.1	– 34.7	– 34.4	– 34.7	– 35.4	– 34.7	– 35.7	– 35.7	–	–	–
31	– 32.1	– 33.1	– 33.4	– 33.8	– 34.5	– 33.3	– 34.5	– 34.3	– 34.9	– 34.2	– 34.7	– 34.4	– 34.1	– 34.7	–	–	–	–	–
32	– 31.6	– 32.5	– 33.5	– 33.9	– 33.2	– 32.6	– 34.4	– 32.9	– 33.9	– 33.9	– 33.8	– 34.3	– 34.5	– 33.8	– 34.3	–	–	–	–
33	– 32.5	– 27.5	– 28.9	– 28.5	– 29.3	– 27.3	– 30.6	– 29.1	– 28.9	– 29.3	– 29.3	– 30.5	– 29.6	– 29.7	– 30.1	– 29.8	– 30.7	– 30.4	–
34	– 32.3	– 34.0	– 34.4	– 34.3	– 34.9	– 34.3	– 35.1	– 33.6	– 35.4	– 35.5	– 34.9	– 35.0	– 35.0	– 34.8					–
35	– 31.1	–	–	–	–	–	–	–	–	–	–	–	–	–	–	–	–	–	–
36	– 31.3	–	–	–	–	–	–	–	–	–	–	–	–	–	–	–	–	–	–

*n-C_x_ denotes normal alkane where x refers to carbon number; Pr and Ph denote pristane and phytane, respectively.

### 3.3 Molecular, stable carbon and hydrogen isotopic compositions of natural gases

Both hydrocarbon and non-hydrocarbon gases are detected in the natural gas samples in this study. Most samples are dominated by methane, with the proportions of 46.89–84.18%, whereas methane in one sample from the drill-hole S5-11 only accounts for 12.57% (Table 6). The ethane and propane proportions are range from 2.43 to 20.92% and from 0.41 to 21.56%, respectively (Table 6). The CO_2_ is the most common non-hydrocarbon species in the studied samples, with most samples containing CO_2_ in 2.70–11.01%, whereas there are two samples from S5-9 and S5-11 having abnormally high CO_2_ contents, which range from 30 to 48% (Table 6). Besides, there are also trace amounts of H_2_ and N_2_, with their proportions in the ranges of 0–0.76% and 0–1.85%, respectively (Table 6).

The δ^13^C ratios of methane, ethane, and propane range from − 49.4‰ to – 44.7‰, from − 39.0‰ to – 32.5‰, and from − 34.1‰ to – 28.2‰, respectively (Table 6). The studied samples display normal patterns regarding δ^13^C ratios of methane, ethane, and propane, i.e. δ^13^C_1_ < δ^13^C_2_ < δ^13^C_3_ (Table 6). The δD ratios of methane, ethane, and propane are in the ranges of – 217‰ to – 162‰, – 173 to – 111‰, and − 133‰ to – 105‰, respectively (Table 6).

**Table 6:** Molecular, stable carbon, and hydrogen isotopic compositions of oil-associated natural gases.

Item	Drillhole	S7	S1	S1-8	S1-9	S1CX	S5-9	S5-12	S1-1
	Sample ID	G1	G2	G3	G4	G5	G6	G7	G8
	Depth/m	7568	7321	7342	7373	7405	7648	7414	7658
	Age	O	O	O	O	O	O	O	O
Mole proportions/%	H_2_	0.76	0.32	0.54	0.02	0.17	0.19	0.02	0.00
	O_2_	0.00	0.00	0.00	0.00	0.00	0.00	0.00	0.00
	N_2_	1.85	1.55	1.81	1.12	1.82	0.00	0.00	1.49
	CO	0.00	0.00	0.00	0.00	0.00	0.00	0.00	0.00
	CO_2_	6.90	11.01	10.43	3.59	6.87	47.80	30.00	2.70
	CH_4_	46.89	84.18	74.04	67.79	77.37	47.57	12.57	82.89
	C_2_H_6_	20.92	2.43	7.82	10.39	8.15	2.96	17.29	7.57
	C_2_H_4_	0.00	0.00	0.00	0.00	0.00	0.00	0.00	0.00
	C_3_H_8_	14.78	0.41	3.38	8.16	3.55	0.81	21.56	3.43
	C_3_H_6_	0.00	0.00	0.00	0.00	0.00	0.00	0.00	0.00
	*i*-C_4_H_10_	1.41	0.03	0.60	2.37	0.65	0.12	3.25	0.69
	*n*-C_4_H_10_	4.92	0.06	0.92	4.16	0.97	0.32	10.82	0.91
	C_4_H_8_	0.00	0.00	0.00	0.00	0.00	0.00	0.00	0.00
	*i*-C_5_H_12_	0.59	0.00	0.22	1.19	0.22	0.08	1.52	0.19
	*n*-C_5_H_12_	0.97	0.00	0.22	1.21	0.22	0.15	2.97	0.14
δ^13^C/‰	CO_2_	– 14.0	–	–	–	–	– 2.3	– 2.5	– 6.7
	CH_4_	– 48.4	– 44.7	– 47.2	– 46.6	– 46.0	– 49.4	– 49.2	– 46.7
	C_2_H_6_	– 39.0	– 33.1	– 33.8	– 34.1	– 34.4	– 34.3	– 38.6	– 37.9
	C_3_H_8_	– 33.9	– 30.8	– 31.2	– 31.9	– 32.1	– 31.7	– 34.1	– 33.3
	*i*-C_4_H_10_	– 33.6	– 31.3	– 31.9	– 32.1	– 32.4	– 32.1	– 33.7	– 32.3
	*n*-C_4_H_10_	– 32.0	– 29.8	– 30.7	– 31.1	– 31.4	– 31.7	– 32.3	– 31.7
	*i*-C_5_H_12_	–	– 30.0	–	–	–	– 31.8	– 31.6	– 30.5
	*n*-C_5_H_12_	–	– 30.2	–	–	–	– 32.0	– 31.9	– 31.6
δD/‰	CH_4_	–	– 174	– 169	– 168	– 162	– 181	– 217	– 217
	C_2_H_6_	–	– 129	– 111	– 119	– 111	–	– 173	– 173
	C_3_H_8_	–	– 116	–	– 107	– 105	–	– 133	– 133
	*i*-C_4_H_10_	–	–	–	– 103	– 106	–	– 107	– 107
	*n*-C_4_H_10_	–	–	–	– 95	– 96	–	– 120	– 120
	*i*-C_5_H_12_	–	–	–	– 103	– 95	–	– 95	– 103
	*n*-C_5_H_12_	–	–	–	– 84	– 77	–	– 108	– 108
R_methane_ (%)		0.57	1.19	0.77	0.87	0.97	0.40	0.43	0.85
T_methane_/^o^C		80	174	118	134	148	35	45	132
R_ethane_ (%)		1.20	2.51	2.36	2.29	2.22	2.24	1.29	1.44
T_ethane_/^o^C		175	270	262	258	254	256	185	199

*O denotes Ordovician; R_methane_ and T_methane_ denote VR and formation temperature estimated based on methane δ^13^C ratios; R_ethane_ and T_ethane_ denote VR and formation temperature estimated based on ethane δ^13^C ratios. The R_methane_ and R_ethane_ is calculated with the equations of R_methane_=(51.8 + δ^13^C_methane_)/5.99 and R_ethane_=(44.4 + δ^13^C_ethane_)/4.50, respectively according to the reference^49^. Both T_methane_ and T_ethane_ values are calculated with the equation of T=(Ln(VR) + 1.19)/0.00782 according to the reference^50^.

### Bitumen reflectance characteristics

Solid bitumen reflectance (V_b_) values are variable and can be classified into two groups (Table 7). One group of solid bitumen has relatively lower reflectance values, which range from 1.05 to 1.77%, whereas the other group of solid bitumen has higher reflectance values (2.17–2.20%; Table 7). The VR of solid bitumen is calculated according to the conversion formula^51^, VR = 0.938*V_b_ + 0.3145. The VR values for the Group 1 solid bitumen and Group 2 solid bitumen are in the ranges of 1.30–1.97% and 2.35–2.38%, respectively. The formation temperatures for Group 1 solid bitumen are estimated by the conversion formula for burial heating conditions, T=(Ln(VR) + 1.68)/0.0124, and formation temperatures for Group 2 solid bitumen are estimated by conversion formula for hydrothermal systems, T=(Ln(VR) + 1.19)/0.00782, considering their much higher reflectance values^50^. Temperatures for solid bitumen from Group 1 and Group 2 range from 157 to 190 °C and from 252 to 254 °C, respectively (Table 7).

**Table 7:** Reflectance values of solid bitumen and estimated formation temperatures.

Drillhole	Depth/m	Age	Morphology	Rb/%	VR/%	Temperature/^o^C
S7	6566.18	O	Disseminated bitumen	1.05	1.30	157
S7	6834.67	O	Disseminated bitumen	1.77	1.97	190
S7	6877.9	O	Disseminated bitumen	1.14	1.38	162
S1-3	7265.85	O	Disseminated bitumen	1.51	1.73	180
S1-3	7265.85	O	Disseminated bitumen	1.68	1.89	187
S1-3	7269.85	O	Bitumen nodules	2.17	2.35	252
S1-3	7269.85	O	Bitumen nodules	2.20	2.38	254

*O denotes Ordovician; Rb and VR denote bitumen reflectance and vitrinite reflectance equivalent, respectively.

### Fluid inclusion thermometry features in calcites

**Fig. 6:**
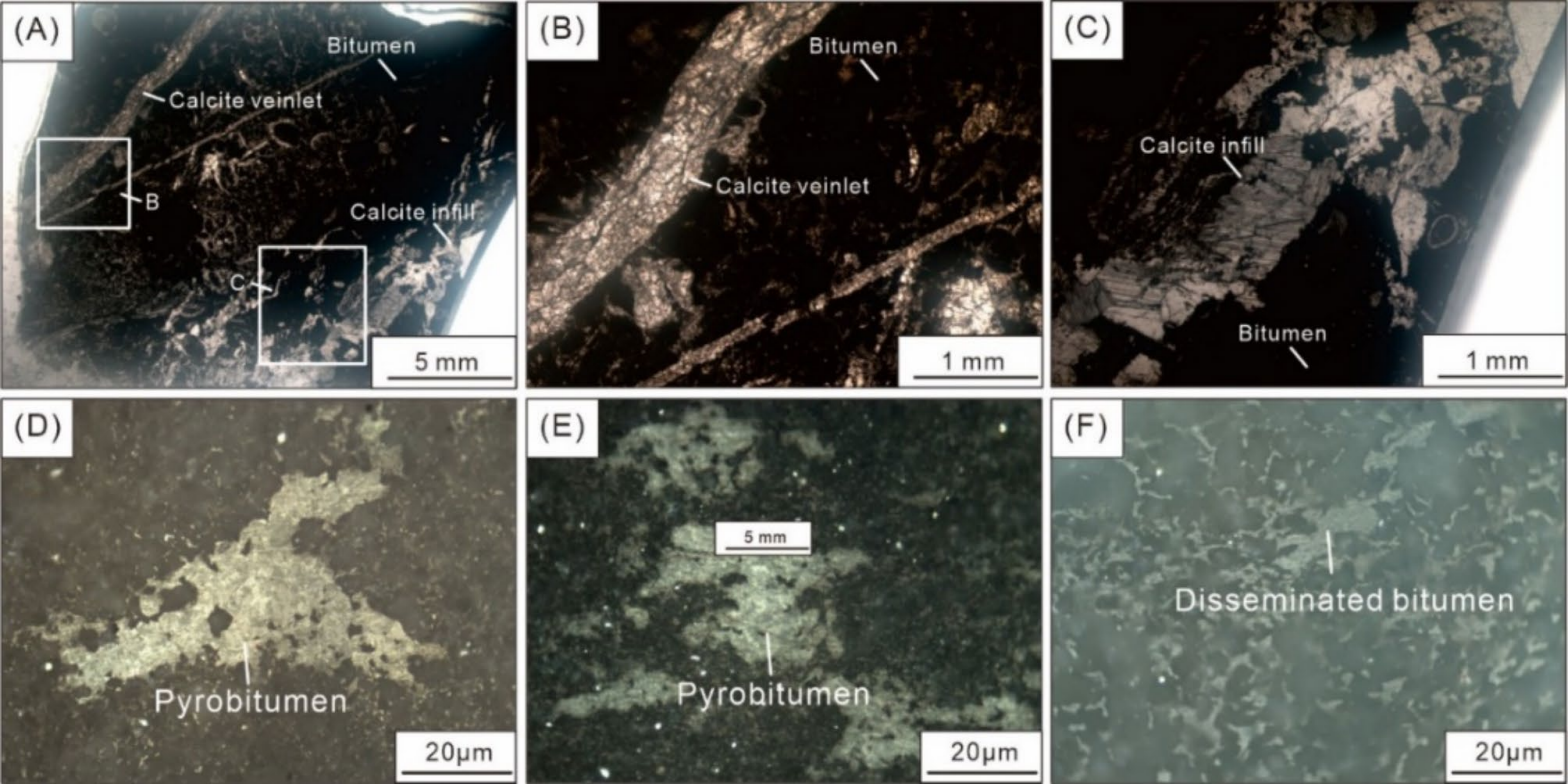
Typical optical photomicrographs of distribution of bitumen and calcite veinlets and infills (A–C), and bitumen morphologies (D–F).

**Fig. 7:**
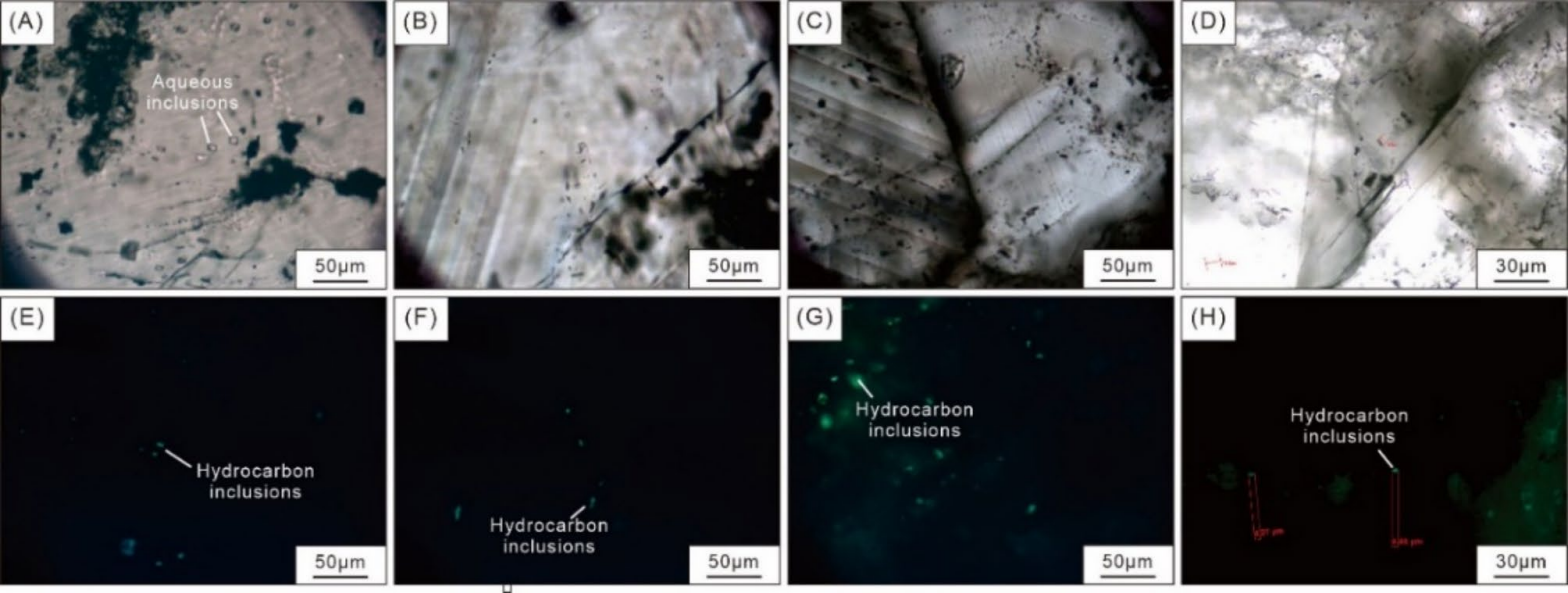
Typical optical photomicrographs of fluid inclusions from calcites in the studied samples (A–D), and hydrocarbon inclusions are identified with UV lights (E–H).

The studied calcites are associated with solid bitumen and either display as veinlets or fill the fractures and pores of the matrix (Fig. 6A and F), and both types of calcites contain abundant oil and aqueous inclusions (Fig. 7A and H). Most oil inclusions comprised two phases at room temperature in the calcite samples (Fig. 7A and H). Oil inclusions are mainly 4–17 μm and are yellow under fluorescence light, and can be regarded as primary oil inclusions (Fig. 7E and H). These oil inclusions display elliptical shapes and are randomly distributed as isolated occurrences within the crystal (Fig. 7A and H). In petroliferous basins, reservoir pore water has soluble hydrocarbon concentrations close to or at saturation. Aqueous inclusions coeval with oil inclusions, were assumed to be gas-saturated and could record the true temperature of fluid entrapment without any pressure correction^52^. Homogenization temperatures of the aqueous inclusions, which coexisted with oil inclusions, ranged from 95.6  to 154.7 °C (average is 123.9 °C; Table 8).

**Table 8:** Homogenization temperatures of fluid inclusions in calcite veinlets within the ordovician reservoirs.

Drillhole hole	Depth/m	Type	UV color	Size/µm	Th/°C	Salinity%NaCl	Density
SB1-3	7261	Aqueous	None	9.5	131	10.1	1.00
SB1-3	7261	Aqueous	None	8.2	133	10.0	1.00
SB1-3	7261	Aqueous	None	5.9	122	–	–
SB1-3	7261	Aqueous	None	9.9	129	7.4	0.99
SB1-3	7261	Aqueous	None	6.8	137	–	–
SB1-3	7261	Aqueous	None	5.8	123	3.3	0.97
S7	7258	Aqueous	None	5.1	120	–	0.94
S7	7258	Aqueous	None	4.5	125	–	0.94
S7	7258	Aqueous	None	5.5	130	–	0.93
S7	7258	Aqueous	None	5.1	129	–	0.94
S7	7258	Aqueous	None	7	115	17.1	1.07
S7	7258	Aqueous	None	11.7	117	17.9	1.07
S7	7258	Aqueous	None	6.7	110	–	0.95
S7	7258	Aqueous	None	5.5	113	–	0.95
S7	7258	Aqueous	None	6.7	111	–	0.95
S7	7258	Aqueous	None	6.3	154	–	0.91
S7	7258	Aqueous	None	4.9	147	–	0.92
S7	7258	Aqueous	None	4.4	155	–	0.91
S7	7258	Aqueous	None	6.3	96	–	0.96
S7	7258	Aqueous	None	17.3	112	5.2	0.99
S7	7258	Aqueous	None	6	104	–	0.96
S7	7258	Aqueous	None	14.1	115	13.6	1.04
S7	7258	Aqueous	None	6.5	122	11.9	1.03
S7	7258	Aqueous	None	17.3	108	10.9	1.03
S7	7258	Aqueous	None	11.6	116	8.0	1.00
S7	7258	Aqueous	None	16.1	113	10.7	1.02
S7	7258	Aqueous	None	9.1	114	–	0.95
S7	7258	Aqueous	None	6	127	–	0.94
S7	7258	Aqueous	None	5.5	124	–	0.94
S7	7258	Aqueous	None	4.9	133	–	0.93
S7	7258	Aqueous	None	6.7	125	–	0.94
S7	7258	Aqueous	None	7.7	129	–	0.94
S7	7258	Aqueous	None	16	124	–	0.94
S7	7258	Aqueous	None	7.9	127	6.8	0.99
S7	7258	Aqueous	None	9.7	129	–	0.94
S7	7258	Aqueous	None	5.8	130	6.1	0.98
S7	7258	Aqueous	None	6.6	128	6.4	0.98
S7	7258	Aqueous	None	6.5	134	7.8	0.99
SB1-3	7261	Oil	Yellowish	9	81	–	–
SB1-3	7261	Oil	Yellowish	5.8	79	–	–
SB1-3	7261	Oil	Yellowish	7.2	79	–	–
SB1-3	7261	Oil	Yellowish	8.9	75	–	–
SB1-3	7261	Oil	Yellowish	8.2	74	–	–
SB1-3	7261	Oil	Yellowish	9.1	72	–	–
SB1-3	7261	Oil	Yellowish	7.4	60	–	–
SB1-3	7261	Oil	Yellowish	7.8	74	–	–
SB1-3	7261	Oil	Yellowish	11.5	53	–	–
SB1-3	7261	Oil	Yellowish	7.9	85	–	–
SB1-3	7261	Oil	Yellowish	8.9	80	–	–
SB1-3	7261	Oil	Yellowish	8	61	–	–
SB1-3	7261	Oil	Yellowish	8.9	55	–	–
S7	7258	Oil	Yellowish	5.5	79	–	–
S7	7258	Oil	Yellowish	4.6	84	–	–
S7	7258	Oil	Yellowish	11	93	–	–
S7	7258	Oil	Yellowish	8.5	103	–	–
S7	7258	Oil	Yellowish	7.7	93	–	–
S7	7258	Oil	Yellowish	7.4	105	–	–

*Th denotes homogenization temperature.

## Discussions

### Homogeneous oil source suggested by uniform hydrocarbon molecular proxies

**Fig. 8:**
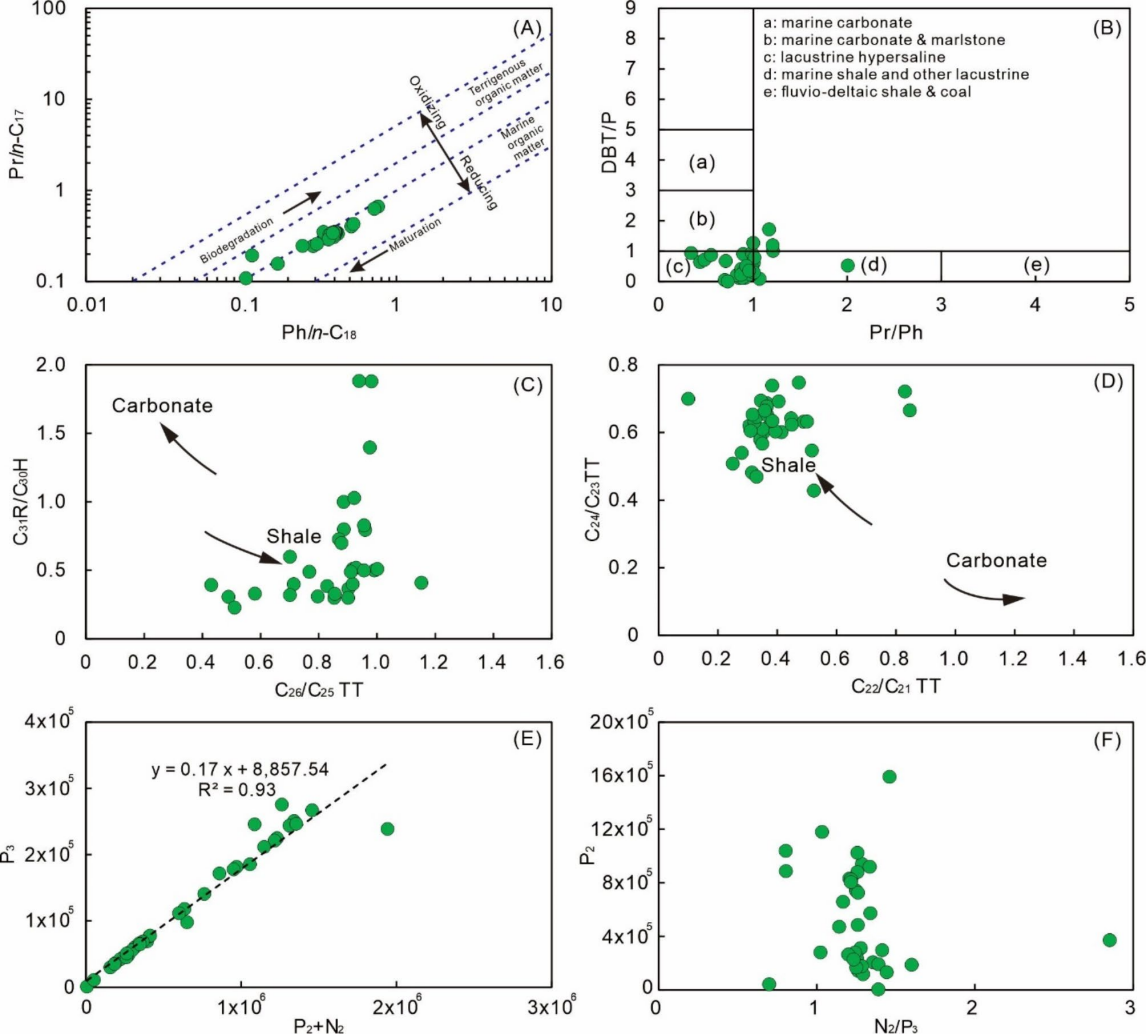
Empirical diagrams for biomarker and light hydrocarbon ratios of the studied samples: (A) Pr/*n*-C_17_ vs. Ph/*n*-C_18_ (after references^54,55^); (B) DBT/P vs. Pr/Ph (after reference^56^); (C) C_31_R/C_30_H vs. C_26_/C_25_ TT, H and TT denote hopane and tricyclic terpane, respectively (after reference^57^); (D) C_24_/C_23_vs. C_22_/C_21_ TT (after reference^58^); (E) P_3_vs. P_2_ + N_2_, integration area of specific light hydrocarbons; (F) P_2_vs. N_2_/P_3_.

Pr and Ph are two commonly used compounds for understanding redox conditions of depositional environments for source rocks and oil-source correlations. Both Pr and Ph are originally derived from phototrophic organisms and purple sulfur bacteria, whereas Ph is generally formed under more reducing conditions than Pr^53^. Thus, oils with low Pr/Ph ratios (< 1) commonly suggest that their source rocks were formed under reducing conditions^53^. In this study, samples commonly have Pr/Ph in 0.5–2.0 (average is 0.92), suggesting that source rocks for crude oils are formed under weakly-oxidizing to reducing conditions^53^. The binary plot of Pr/*n*-C_17_ alkane vs. Ph/*n*-C_18_ alkane is widely used for identifying depositional features of source rocks and potential influences of post-accumulation processes^54,55^. The Pr/*n*-C_17_-Ph/*n*-C_18_ plot suggests that marine organic matter is the dominant contributor to the studied oils (Fig. 8A). Biomarker compositions of oils may also preserve details about the lithological features of source rocks. For example, binary plots established by TT, for example, C_31_R/C_30_H vs. C_26_/C_25_TT (Fig. 8C) and C_24_/C_23_ TT vs. C_22_/C_21_TT (Fig. 8D), suggest that shales may be the dominant lithological type of source rocks for the studied oils; the binary plot of DBT/P vs. Pr/Ph shows that the majority of the samples are located in the region of “lacustrine hypersaline”, which this is a common signal for hydrothermally altered samples (Fig. 8B)^59–62^.

Light hydrocarbons may also preserve useful information about the origin of the studied samples (Fig. 4M and O). Several parameters were established based on light hydrocarbons to determine oil sources, such as K_1_, K_2_, P_2_, P_3_, and N_2_^63–65^. Samples derived from the same source rocks generally have rigorous positive correlations between P_3_ and (P_2_ + N_2_) values^63–65^. The regression of P_3_ and (P_2_ + N_2_) values for the studied samples yield a positive trend with the coefficient R^2^ of 0.93 (Fig. 8E), supporting that samples in this study were derived from the same source rock. Besides, the studied samples are also located in similar regions of the binary plot of P_2_ vs. N_2_/P_3_ (Fig. 8F), providing additional evidence that the studied samples are generated by the same source rocks^63–65^.

**Fig. 9:**
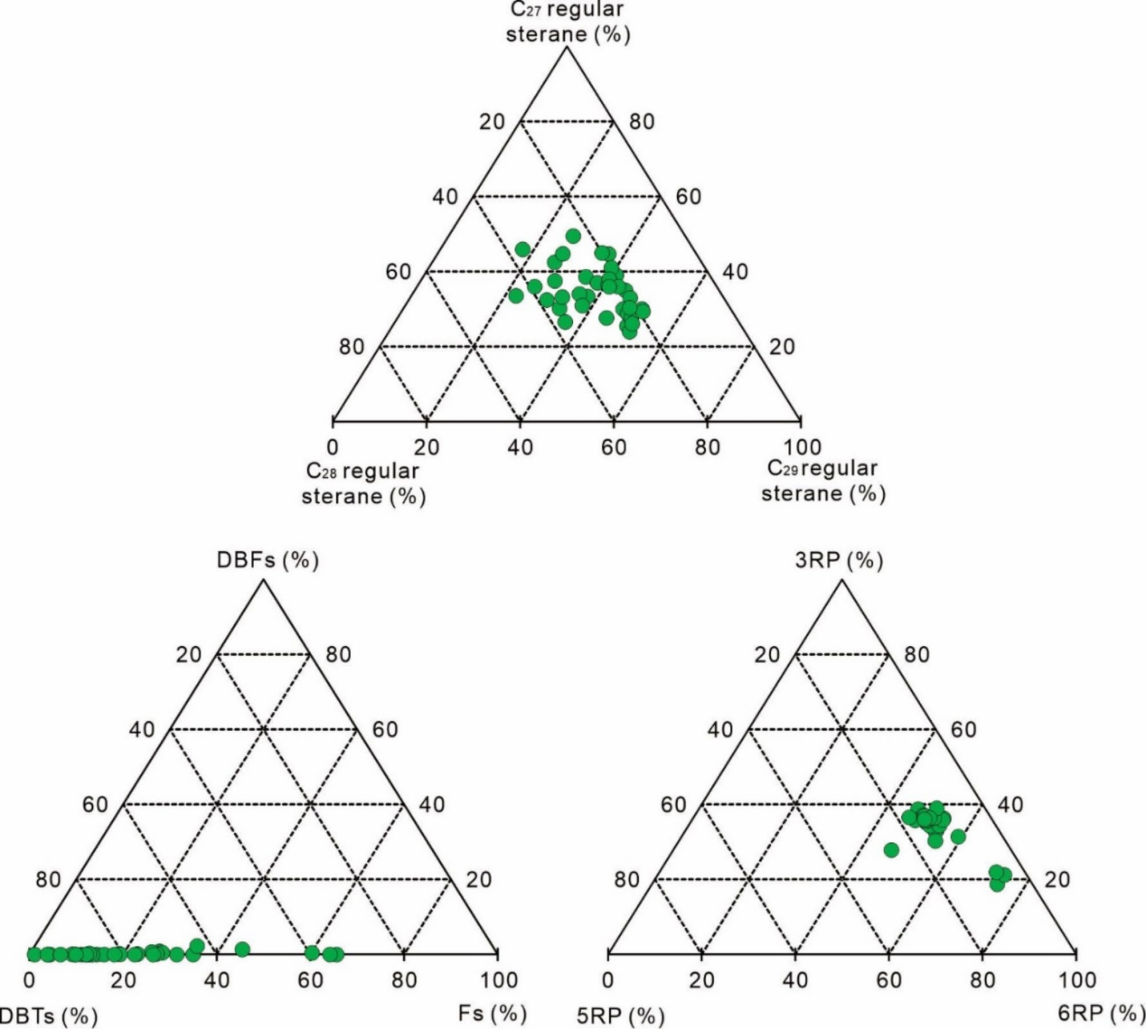
Ternary plots of relative proportions of C_27_-C_28_-C_29_ regular sterane, DBFs-DBTs-Fs (dibenzothiophenes, dibenzofurans, and fluorenes), and 3RP-5RP-6RP.

In addition, there are several ternary plots of specific hydrocarbon species proposed to classify oils, such as C_27_-C_28_-C_29_ regular steranes, dibenzothiophene-dibenzofuran-fluorene (DBTs-DBFs-Fs), and isoalkanes-cyclopentanes-cyclohexanes (3RP-5RP-6RP)^63–66^. The samples span over a wide range of ratios in these ternary plots (Fig. 9), and such features may reflect local variations in source rock character or post-accumulation effects^63–66^.

### Thermogenic genesis of gaseous hydrocarbons associated with oil reservoirs

**Fig. 10:**
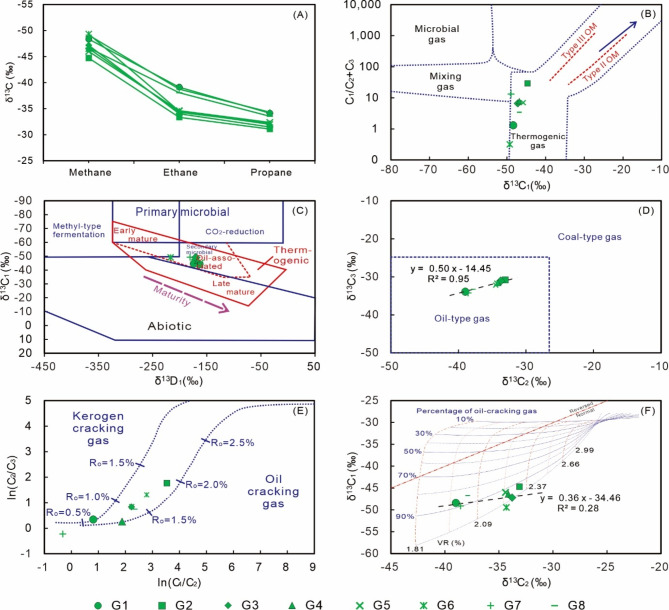
Genetic diagrams for identifying the origin of natural gases. (A) δ^13^C values of methane, ethane, and propane; (B) C_1_/(C_2_ + C_3_) vs. δ^13^C_1_ (after reference^69^); (C) δ^13^C_1_vs. δD_1_ (after reference^70^); (D) δ^13^C_3_vs. δ^13^C_2_ (after reference^71^); (E) ln (C_2_/C_3_) vs. ln (C_1_/C_2_) (after reference^67^); (F) δ^13^C_1_vs. δ^13^C_2_ (after reference^72^). C_1_, C_2_, and C_3_ denote proportions of methane, ethane, and propane, respectively. δ^13^C_1_, δ^13^C_2_, and δ^13^C_3_ denotes the stable carbon isotopic ratios (δ^13^C values) of methane, ethane, and propane, respectively. δ^13^D_1_ denotes the stable hydrogen isotopic ratio (δ^13^D value) of methane.

Oil reservoirs in the studied area are generally associated with natural gases, and understanding the gas genesis may provide useful information for constraining the hydrocarbon accumulation processes^67,68^. The studied natural gas samples have consistent, normal distribution patterns of δ^13^C values of methane, ethane, and propane (Fig. 10A), suggesting that the studied hydrocarbon gas samples may have been generated by similar processes.

In hydrocarbon-bearing basins, there are mainly two types of hydrocarbon gases, including biogenic gas and thermogenic gas^70^. Previous studies show that biogenic gas is commonly characterized by much more depleted methane δ^13^C values and higher methane contents compared to thermogenic gas^73^. The binary plot of C_1_/(C_2_ + C_3_) vs. δ^13^C_1_ is thus proposed to distinguish biogenic gas, thermogenic gas, and a combination of both^69^. The studied samples belong to thermogenic gases according to the plot of C_1_/(C_2_ + C_3_) vs. δ^13^C_1_ (Fig. 10B). The thermogenic genesis of gas samples is also supported by the binary plot of δ^13^C_1_ vs. δ^13^D (Fig. 10C)^70^. Kerogen, crude oil, and coal are common precursors of thermogenic hydrocarbon gases in basins^74^. Coal-derived gases (i.e. “coal-type gas”) generally have more enriched δ^13^C_2_ and δ^13^C_3_ ratios compared to those decomposed from kerogen and oil (i.e. “oil-type gas”, which is generated from decomposition of sapropelic organic matter (type I and II kerogen), including gases from direct decomposition of sapropelic rocks and secondary cracking of crude oil)^69^, thus the binary plot of δ^13^C_2_vs. δ^13^C_3_ is proposed to identify the specific precursors for gases^69^. The studied samples are oil-type gases according to the δ^13^C_2_vs. δ^13^C_3_ plot, suggesting that gases are generated by thermal decomposition of kerogen and crude oil (Fig. 10D).

Gases generated via the thermal decomposition of kerogen and crude oil can be distinguished by relative proportions of ethane, methane, and propane^75^. Methane is generated faster than ethane and propane during kerogen cracking, whereas the opposite scenario occurs during oil cracking^76,77^. The binary plot of ln (C_2_/C_3_) vs. ln (C_1_/C_2_) is thus proposed to distinguish oil-cracking gas and kerogen-cracking gas^67^. Both oil cracking and kerogen cracking processes contribute to the studied samples according to the plot of ln (C_2_/C_3_) vs. ln (C_1_/C_2_) (Fig. 10E). The binary plot of δ^13^C_1_vs. δ^13^C_2_ also supports a mixed contribution of oil-cracking gas and kerogen-cracking gas, with oil cracking being the main process responsible for the studied gas samples (Fig. 10F).

### Incongruent thermal maturity levels assessed by multiple molecular proxies

Molecular compositions of crude oils are modified with increased thermal maturity levels, and many useful molecular proxies have been established in previous studies to assess the maturity levels of crude oils^78–82^.

**Fig. 11:**
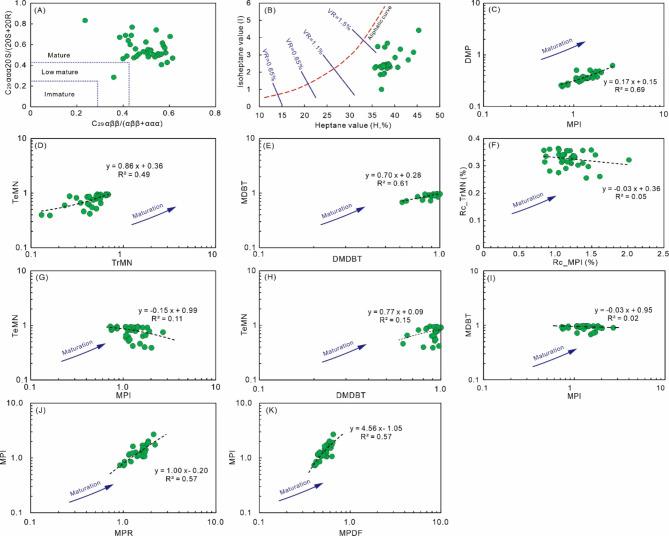
Binary diagrams for molecular maturity indicators. (A) C_29_sterane ααα20S/(20 S + 20R) vs. C_29_sterane αββ/(αββ + ααα) (after reference^86^); (B) isoheptane value vs. heptane value (after reference^85^); (C) DMP vs. MPI; (D) TeMN vs. TrMN; (E) MDBT vs. DMDBT; (F) Rc_TrMN vs. Rc_MPI, Rc denote VR calculated based on TrMN and MPI, respectively; (G) TeMN vs. MPI; (H) TeMN vs. DMDBT; (I) MDBT vs. MPI; (J) MPI vs. MPR; (K) MPI vs. MPDF.

The studied samples have broadly homogeneous sterane and terpane maturity proxies^26,48,55,83^, such as C_31_ 22 S/(22 S + 22R)H (0.2–0.6, average is 0.5), C_30_moretane/C_30_H (0.1–0.5, average is 0.2), Ts/(Ts + Tm) (0.2–0.8, average is 0.5), C_29_Ts/(C_29_Ts + C_29_H) (0.1–0.6, average is 0.3), C_29_sterane ααα20S/(20 S + 20R) (0.3–0.8, average is 0.5), and C_29_sterane αββ/(αββ + ααα) (0.2–0.6, average is 0.5) (Table 3; Fig. 11A). These features suggest that the studied samples are mature oils. It is noted that many samples have C_29_sterane ααα20S/(20 S + 20R) higher than the equilibrium value of 0.55 (Table 3; Fig. 11A), and such features may be caused by post-accumulation processes, such as biodegradation, mixing of oils with different maturities and origins^25,83,84^. The coexistence of UCM hump and *n*-alkanes in the studied samples suggests that paleo-biodegradation and multiple oil accumulation processes may have occurred, and thus both processes may be responsible for the abnormally high C_29_sterane ααα20S/(20 S + 20R) ratios^25,83,84^.

Light hydrocarbon parameters may also provide useful information about thermal maturity levels, such as 2,4-/2,3-DMP, *n*-C_7_alkane/MCH, H values, and I values^85^. The studied samples have uniform light hydrocarbon maturity parameters, with 2,4-/2,3-DMP, *n*-C_7_alkane/MCH, H value, and I values are in the ranges of 0.24–0.67 (average is 0.35), 1.22–2.62 (average is 1.79), 36–45 (average is 38), and 1.00–4.42 (average is 2.51; Table 4), respectively. The binary plot of I values vs. H values suggests that the studied samples have VR over 1.1% (Fig. 11B)^85^, supporting that oils were generated at the advanced stage of oil window maturity^82^.

Aromatic hydrocarbons also preserve valuable details about thermal maturity levels, especially for highly-matured samples^47^. In general, aromatic parameters based on the same homologous series display positive linear correlations, such as DMP vs. MPI (Fig. 11C), TeMN vs. TrMN (Fig. 11D), and MDBT vs. DMDBT (Fig. 11E). However, the correlations between parameters based on different aromatic homologous series are weak (Fig. 11F and I). The VR values for the studied samples calculated based on TrMN and MPI values are inconsistent (Fig. 11I). The VR_TrMN_ values of samples (0.26–0.36%) are much lower compared to those calculated from MPI values (VR_MPI_ = 0.84–2.01%; Fig. 11H). Indeed, the VR_TrMN_ values (0.26–0.36%) may not reflect the real thermal maturity levels of samples because such low values commonly represent the “pre-oil stage”^82^, and this is not the case in the studied area where abundant oils have been generated and accumulated^26^. It has been reported that the TrMN can display “retrograde” behaviour at advanced maturity levels (i.e. the value decreased with the rise of maturity levels)^47^. This may be responsible for the low VR_TrMN_ values of the studied samples. Although VR_MPI_ values approximate thermal maturity levels of samples, it is noted that the conversion of MPI into VR is based on an equation initially developed on coals and type III kerogens^47^. This may bring uncertainties to VR_MPI_ values in the studied area because oils in the studied area were commonly derived from marine type II source rocks^21^. Thus, uncertainties associated with the conversion equation may partly account for the discrepancies in thermal maturities observed between different parameters. Besides, the MPI combines a stable compound (phenanthrene) with the less stable methylphenanthrenes 1-MP and 9-MP in the denominator^87^, and factors other than thermal maturation may result in anomalously high MPI values, such as water-washing, because of the preferential alteration of phenanthrene compared with methylphenanthrenes^88^. In this study, MPI values positively correlate with methylphenanthrene ratios (e.g. MPR, MPDF; Fig. 11J and K), thus it seems that thermal maturation is still the dominant factor controlling MPI values of samples. Such discrepancies in thermal maturity indicators may also be attributed to the variable susceptibility of different homologous series to post-accumulation processes. The presence of Permian magmatic rocks suggests that the studied area has been affected by the Tarim LIP^31^, and this abnormal heating process may alter the thermal regime of the studied area^26^. Different aromatic homologous series may behave differently during this hydrothermal heating process^14,47,89,90^, which further resulted in the incongruent maturity parameters.

### Recognition of hydrothermal infiltration with organic geothermometer

Peculiar organic geochemical proxies are sensitive to ambient temperatures, and their features may provide useful constraints to recognize anomalous high temperatures induced by hydrothermal infiltration in oil reservoirs, such as homogenization temperatures of fluid inclusions, the reflectance of solid bitumen, aromatic compounds, light hydrocarbons, and gaseous hydrocarbons^3,14,15,91^.

**Fig. 12:**
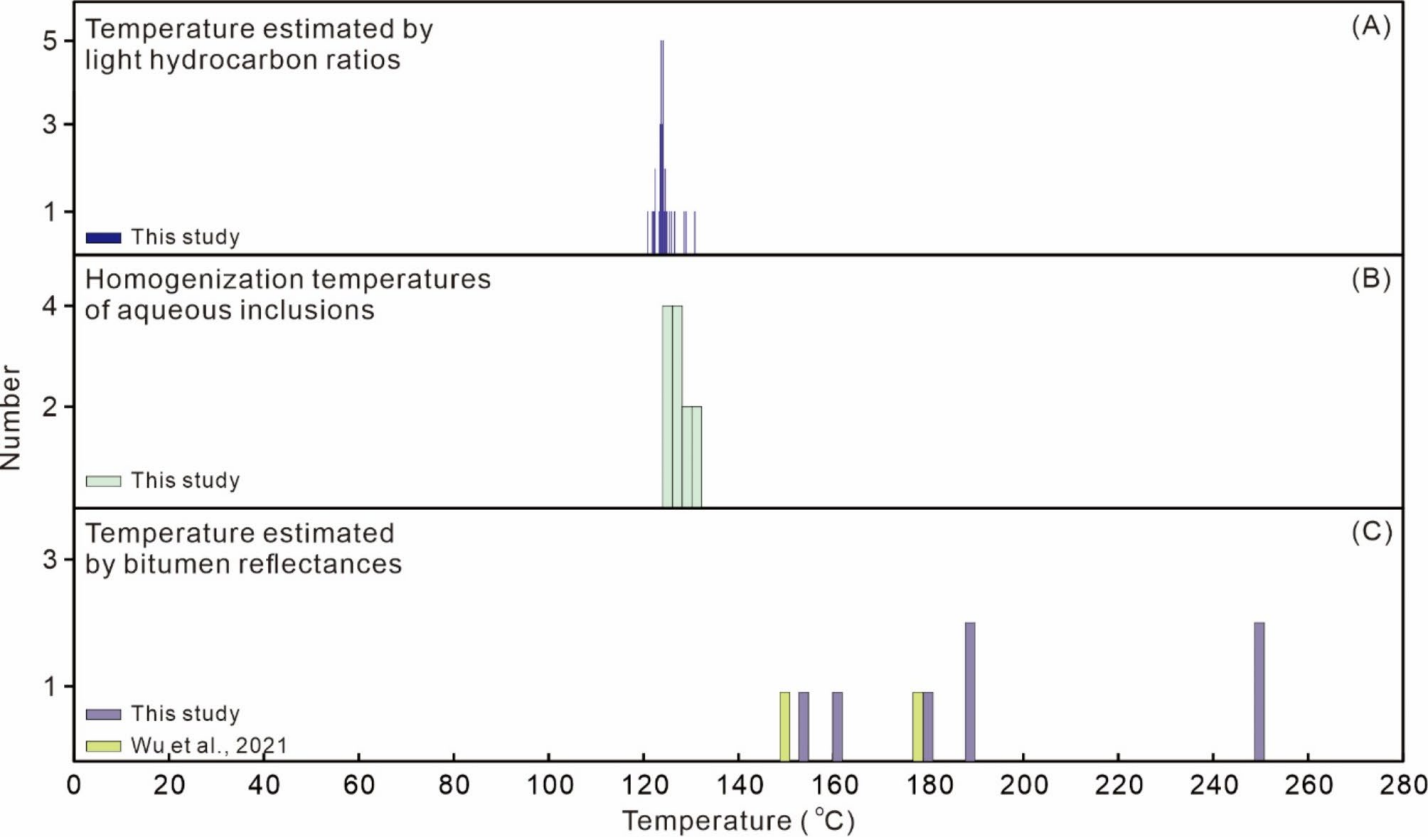
Histograms for temperatures estimated by light hydrocarbon compositions (A), homogenization temperatures of aqueous inclusions coeval with oil inclusions (B), and reflectance of solid bitumen (C).

The stable carbon isotopic compositions of hydrocarbon gases are strongly affected by thermal maturity levels^72^. It is noted that there is a rigorous positive correlation between δ^13^C_2_ and δ^13^C_3_ values (regression coefficient r^2^ = 0.95; Fig. 10D), whereas the positive correlation between δ^13^C_2_ and δ^13^C_1_ is weaker (regression coefficient r^2^ = 0.28; Fig. 10F). Such features may be caused by the different susceptibility of carbon isotopic compositions of methane, ethane, and propane to processes other than thermal maturation^69^. The carbon isotopic ratio of methane is more sensitive to secondary processes or potential mixing with biogenic sources compared to those of ethane and propane^69^. Thus, the weak δ^13^C_1_–δ^13^C_2_ correlation suggest that the carbon isotopic compositions of methane may have been modified by secondary processes or mixing with biogenic gases in the studied area, and the δ^13^C_2_ and δ^13^C_3_ values may be mainly controlled by thermal maturation. The maturity levels of natural gases are evaluated by carbon isotopic ratios of ethane and the VR values are in the range of 1.20–2.51% (Table 6). However, the VR values estimated by δ^13^C_1_ values are in a lower range (0.40–1.19%; Table 6). The formation temperatures of natural gases are estimated according to the conversion formula for hydrothermal systems^50^, T = (ln(VR) + 1.19)/0.00782. The calculated temperatures according to δ^13^C_2_ values are in the range of 175–270 °C (Table 6; Fig. 12A), However, temperatures calculated based on δ^13^C_1_ ratios are much lower (35–174 °C; Table 6; Fig. 12A), and the presence of particularly low temperatures (e.g. 35 °C) suggest that portions of methanes may be formed by processes other than thermal degradation of organic matter (e.g. biogenic processes).

The generation temperatures for light hydrocarbons are estimated according to the conversion formula proposed by Mango (1997)^92^, T = 140 + 15 [ln(2,4-/2,3-DMP)]. The calculated temperatures based on light hydrocarbon ratios range from 119 to 134 °C (average is 124 °C; Fig. 12B). The homogenization temperatures of the aqueous inclusions, which coexisted with oil inclusions, are distributed between 95.6  and 154.7 °C (average is 123.9 °C; Fig. 12C). The temperatures estimated with light hydrocarbons and fluid inclusion thermometry are concordant with temperatures necessary for the formation of crude oils^82^.

Temperatures estimated based on bitumen reflectance are relatively higher compared to those calculated from light hydrocarbons and fluid inclusion homogenization temperatures (Fig. 12A and C). Formation temperatures of solid bitumen is estimated according to the conversion formula for hydrothermal systems, T=(ln(VR) + 1.19)/0.00782^50^, considering potential abnormal heating by hydrothermal infiltrations. Formation temperatures of solid bitumen according to their reflectance values range from 157 to 190 °C and from 252 to 254 °C, respectively. The temperatures for the first group of solid bitumen correspond to high-maturity to overmature stages with respect to hydrocarbon generation^82^. Whereas, the abnormally high temperatures documented by the solid bitumen of the second group may not have resulted from the normal burial process as the Ordovician reservoir in the studied area has not undergone such a high temperature according to numerical modeling results without the consideration of hydrothermal infiltration^26^. Thus, the presence of high-temperature solid bitumen may suggest that hydrothermal fluids have infiltrated the reservoirs in the studied area.

**Fig. 13:**
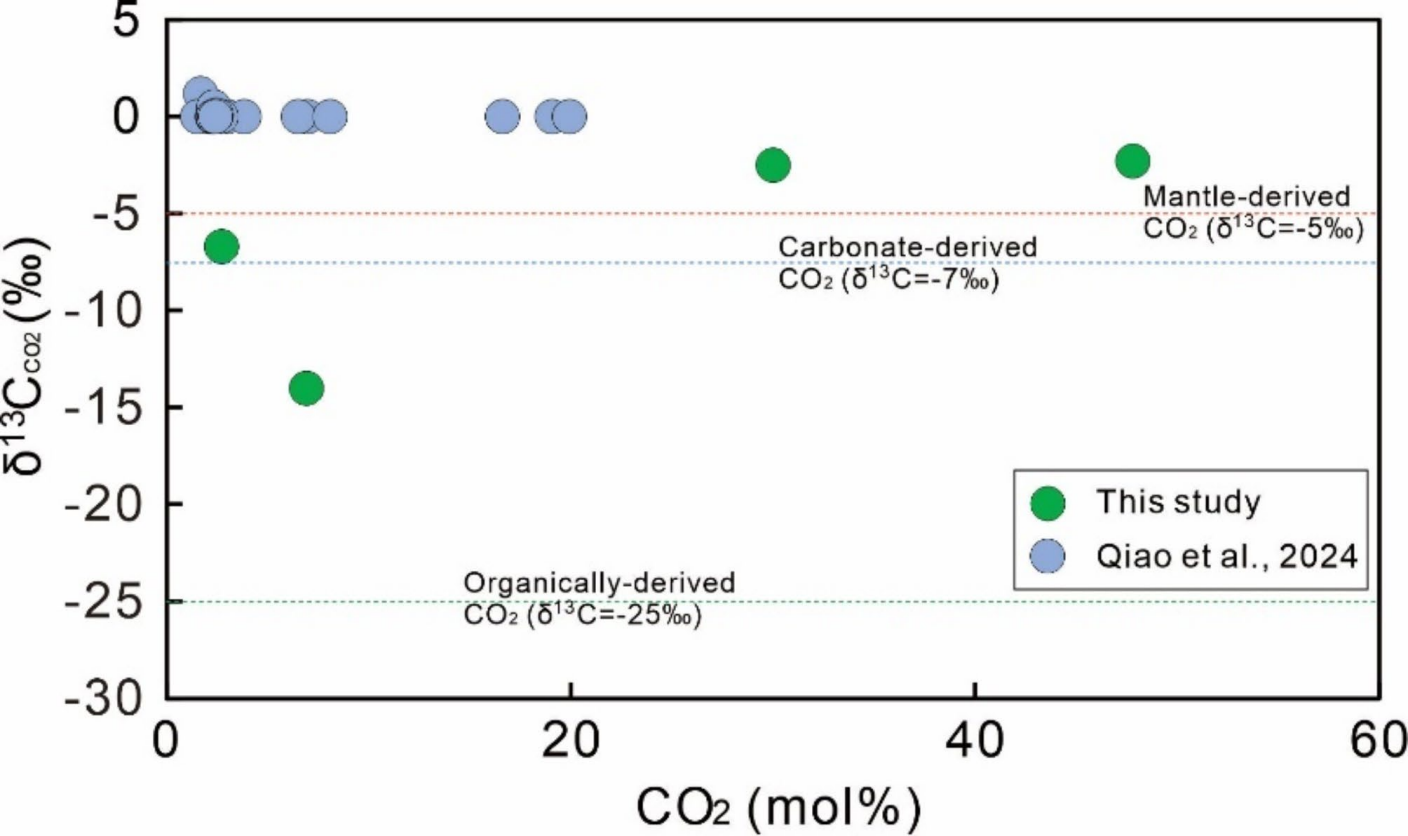
Binary plot of δ^13^C values of CO_2_vs. mole proportions of CO_2_ for identifying the contribution of hydrothermal fluids. Data from reference^26^ are also plotted for comparison purposes.

The occurrence of hydrothermal infiltration in the studied area may also be supported by stable carbon isotopic compositions of CO_2_. CO_2_ is the most common non-hydrocarbon gas species in the studied samples, with the proportions in the range of 2.70–47.80% (Fig. 13). CO_2_ in petroliferous basins may be derived from the thermal decomposition of organic matter (e.g. kerogen, crude oil, and organic acids), dissolution of carbonate minerals (e.g. calcite), and mantle-derived fluids^94–99^. The stable carbon isotopic compositions of CO_2_ generated by different precursors are variable. The δ^13^C values of CO_2_ generated by the organic matter (~ – 25‰) are generally much more depleted compared to those generated via the dissolution of carbonate minerals (~ – 7‰) or derived mantle-sourced fluids (~ – 5‰)^94–99^. Thus, δ^13^C values of CO_2_ can be used to identify whether CO_2_ is derived from organic or inorganic pathways. The δ^13^C values of CO_2_ in the studied samples range from − 14‰ to -2.3‰ (Fig. 13), suggesting that both organic and inorganic processes may have contributed to CO_2_ in the studied samples. It was previously reported that with the increase of temperature, carbonate dissolution occurred and this process can generate large quantities of CO_2_ in deep strata^100^. However, it was found that CO_2_ molar volumes and partial pressures of CO_2_ (i.e. CO_2_ mol%×reservoir pressure) do not increase with the rise of temperatures in the studied area^26^. Thus, carbonate dissolution may not be the dominant factor responsible for the increase in CO_2_ molar volumes and its δ^13^C values in the study area. Therefore, CO_2_ with more enriched δ^13^C values (– 6.7‰ to – 2.3‰) may be derived from mantle-sourced fluids. The presence of mantle-derived CO_2_ supports that hydrothermal fluids infiltrated the studied area.

### Interplay between hydrothermal infiltration and hydrocarbon accumulation in the cratonic region of Northern Tarim basin

The combined outcomes of previous studies and this study show that the Early Permian Tarim LIP-related hydrothermal infiltration may have pervasively affected the cratonic region of the northern Tarim Basin. For example, Lan et al.^101^ discovered ~ 290 Ma hydrothermal calcites in the Yingersu Section of the northern Tarim Basin; Li et al.^2^ and Ge et al.^20^ found that hydrothermal fluid flows may occur in the Tahe Oilfield and Halahatang Oilfield, respectively; this study reveals abnormally high reflectance values of solid bitumen and enriched δ^13^C ratios of CO_2_, coupled with the presence of Permian diabase intrusions^31^, supporting that the Tarim LIP-related hydrothermal infiltration may occur in the Shunbei area^29,102,103^.

Hydrothermally-resultant abnormally high thermal stress may alter the molecular compositions of reservoired crude oils. For example, hydrothermal altered-oils tend to have even over odd carbon number predominance in *n*-alkanes, which may be caused by the preferential oxidization of odd-carbon-number *n*-alkanes or formation of even-carbon-number *n*-alkanes during hydrothermal processes^87^; hydrothermal alteration may also lead to the large variation in biomarker ratios such as Pr/Ph, gammacerane index, and Ts/(Ts + Tm) values of crude oils^59–62,87^; polycyclic aromatic compounds may be formed by the thermal cracking of saturated hydrocarbons with the rapid increase in reservoir temperatures caused by hydrothermal infiltration^14,26^; hydrothermal oils have elevated sulfur, alkanone, and alkylphenol contents, and they also contain residual immature biomarkers and intermediates (such as 17β(H)-hopanes, hopenes, sterenes) and degraded biomarkers (such as Diels’ hydrocarbons, porphyrins with C_27_ max)^14,104–106^; heteroaromatic compounds are enriched in hydrothermally-altered oils^106,107^; hydrothermal fluid have great capacities of dissolving organic compounds from source sediments and thus hydrothermal petroleum preserve alkene contents comparable to those in source rocks^108^. In this study, gammacerane index values span over 0.91–5.24, which may be the result of hydrothermal alteration. The incongruent aromatic maturity parameters may also support that original molecular compositions of organic compounds may have been overprinted by the post-accumulation hydrothermal heating. In addition to modifying the molecular compositions of crude oils, the stable carbon isotopic composition of crude oils may also be affected. For example, abnormal thermal stress caused by hydrothermal infiltration may increase δ^13^C ratios of hydrocarbons^3,26^. In this study, crude oils have homogeneous bulk δ^13^C ratios, which range from − 33.4‰ to – 29.6‰ (average is – 31.9‰), and such negative stable carbon isotopic signatures support that the samples are typical oils derived from marine source rocks^109^. In contrast to the broadly uniform bulk δ^13^C ratios of crude oils, δ^13^C ratios of individual *n*-alkanes display moderate variations (Fig. 5). One sample from S57X has more enriched δ^13^C ratios of *n*-alkanes than other samples (up to 8‰), especially for lower-molecular-weight *n*-alkanes (lower than *n*-C_18_). Natural gas samples from locations near this drill-hole contain much higher proportions of CO_2_ (30–48%) than others and their δ^13^C ratios (between − 2.5‰ and − 2.3‰) exhibit signatures of hydrothermally derived CO_2_ (Table 6). Therefore, the elevated δ^13^C ratios of *n*-alkanes may be caused by abnormal hydrothermal heating.

**Fig. 14:**
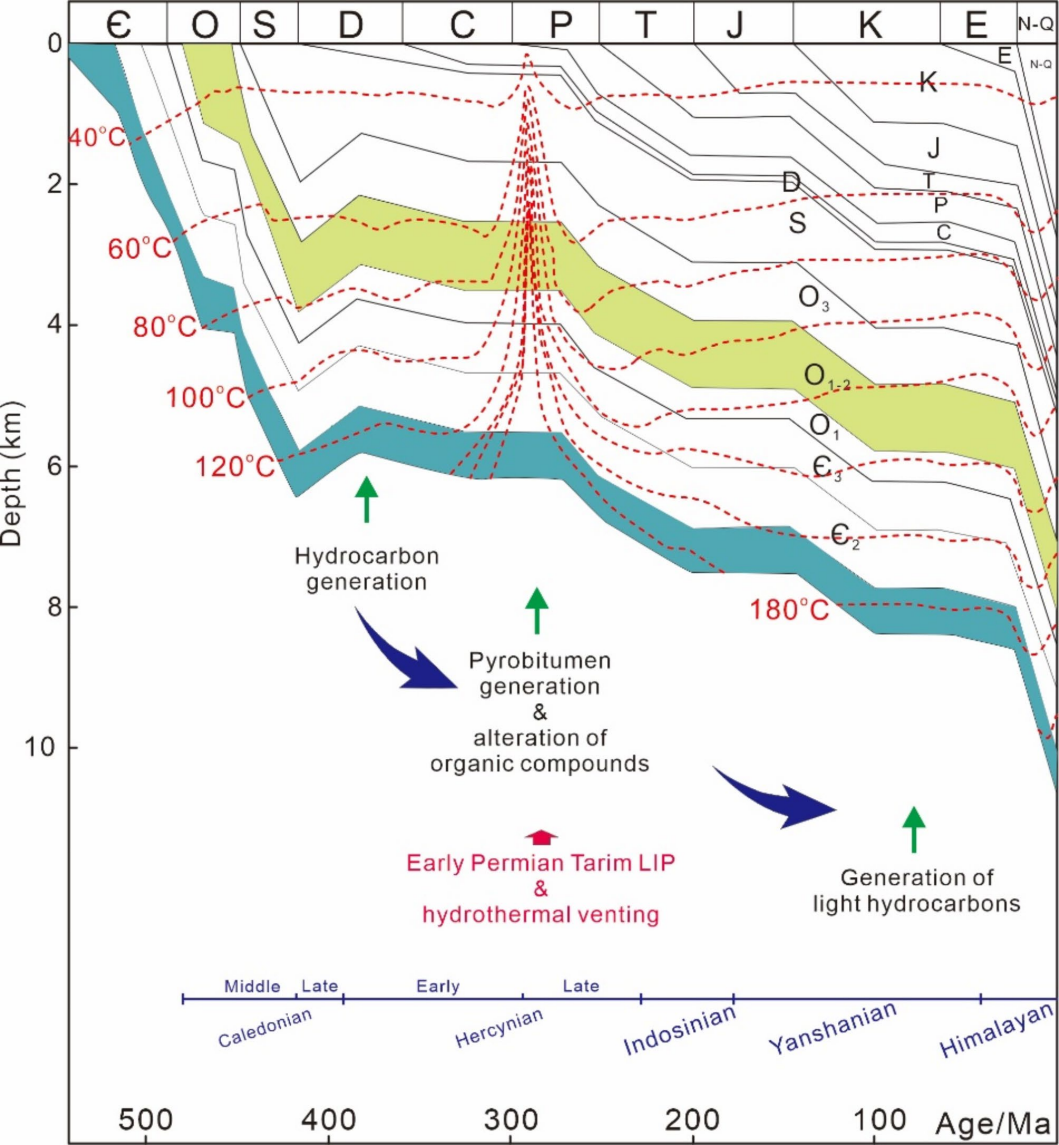
Burial and thermal history modeling results for the Shunbei Oilfied showing the interplay between the Tarim LIP-related hydrothermal infiltration and petroleum accumulation processes in the studied area. Modified after reference^26^.

Deciphering the interplay between hydrothermal infiltration and hydrocarbon accumulation can provide useful information about whether the formation of oil reservoirs is facilitated or destructed by hydrothermal fluid flows. The presence of high-temperature solid bitumen and altered organic compounds in the studied samples suggest that there should have been at least one episode of oil accumulation event prior to the Early Permian Tarim LIP. Indeed, it is suggested that the earliest regional oil accumulation event took place at approximately 399 Ma when the Cambrian source rocks reached the oil-generative peak^21^. Light hydrocarbons are important species in the Shunbei area, especially for condensate samples^26^. The formation temperatures of light hydrocarbons are estimated to be 119–134 °C (average is 124 °C). Light hydrocarbons would be generated during the earliest episode of oil accumulation event according to the local burial history (Fig. 14) if light hydrocarbons are generated directly via the thermal cracking of kerogen. However, light hydrocarbons would be destroyed by the post-dating hydrothermal infiltration considering their strong susceptibilities to thermal stress^92^. Thus, it seems that light hydrocarbons would rather be generated via the thermal cracking of pre-existing crude oils in reservoirs at later stages (Late Yanshanian-Himalayan period) according to their similar formation temperatures (Fig. 14). Besides, heavy fractions (e.g. asphaltenes) precipitate from oils when large quantities of light hydrocarbons are dissolved in oils^110^. Light oil is the main type of oil in the Shunbei area (Fig. 3), thus there may have been an extensive generation of light hydrocarbons and gases from crude oils. This further suggests that hydrothermal heating related to the Tarim LIP may have facilitated the generation and accumulation of pre-existing crude oils. Indeed, oil generation processes that are facilitated by hydrothermal infiltration have been reported both in the northern Tarim Basin and elsewhere worldwide^3^. In addition to providing external heat for source rock maturation, hydrothermal fluids can dissolve abundant transitional metal elements that may have catalytic effects on oil generation^111–113^, and act as a potential driving force improving secondary migration and accumulation of crude oils^114^. Previous studies reveal that organic compounds are often produced, altered and transported in various hydrothermal systems. For example, hydrocarbons were found in modern submarine hydrothermal vent fields, e.g. Guaymas Basin in the Gulf of California^14^, Escanaba Trough, offshore California^115^; organic compounds were widely detected in hydrothermal ore deposits, such as the McArthur River Zn-Pb deposit in the northern Australia^116^, Jinding Zn-Pb deposit in the Yunnan Province, southwestern China^117^; organic compounds were commonly found in hot springs as well (e.g. Tengchong hot spring sinters, Yellowstone Hot Springs, Icelandic hot spring deposits)^59,118–120^. Besides, Finlay et al.^97^ revealed that oils in the United Kingdom North Sea display geochemical features resembling the features of mantle-derived hydrothermal fluids with respect to Re-Os isotopic compositions, CO_2_ and noble gas isotopic compositions, suggesting that oils may have been migrated with hydrothermal fluids. Therefore, hydrothermal fluids may introduce portions of organic compounds to oils in the studied area as well.

## Conclusions

Organic geochemical features of crude oils, solid bitumen, and natural gases, as well as fluid inclusion thermometry, are investigated for the deeply-buried Ordovician carbonate reservoirs in the Shunbei area of northern Tarim Basin, NW China. The studied crude oils are characterized by uniform organic indicators of paraffin, terpanes, steranes, and light hydrocarbons, implying that crude oils are derived from the same source rock. Genetic diagrams such as Pr/*n*-C_17_vs. Ph/*n*-C_18_, DBT/P vs. Pr/Ph, C_31_R/C_30_H vs. C_26_/C_25_TT, and C_24_/C_23_ TT vs. C_22_/C_21_ TT further suggest that marine shales deposited in a reducing-weakly oxidized environment are major source rocks for crude oils. Natural gases are associated with oil reservoirs and are mainly generated via the thermal decomposition of kerogen and crude oil. Solid bitumen with abnormally high reflectance values (2.17–2.20%) occurred in the studied area, suggesting their formation temperatures were in the range of 252–254 ^o^C. The abnormally high temperatures may be caused by hydrothermal infiltration related to the Early Permian Tarim Large Igneous Province. Hydrothermal infiltration is supported by the presence of high contents of CO_2_ (30–48%) with enriched δ^13^C ratios (between − 2.5‰ and − 2.3‰), enriched *n*-alkane δ^13^C ratios, and incongruent temperatures estimated by multiple proxies (e.g. light hydrocarbon compositions, homogenization temperatures of fluid inclusions, and bitumen reflectance). Outcomes of this study suggest that hydrothermal infiltration indeed occurred and may have facilitated hydrocarbon generation in the Shunbei area, and possibly elsewhere in the cratonic regions of the northern Tarim Basin.

## Data Availability

The datasets generated and/or analysed during the current study are available in the [Baidu Netdisk] repository, [https://pan.baidu.com/s/1C6Cwj7ZkjpObbU50CM31EQ?pwd=1234].
